# Interaction between the nasal microbiota and *S. pneumoniae* in the context of live-attenuated influenza vaccine

**DOI:** 10.1038/s41467-019-10814-9

**Published:** 2019-07-05

**Authors:** Wouter A. A. de Steenhuijsen Piters, Simon P. Jochems, Elena Mitsi, Jamie Rylance, Sherin Pojar, Elissavet Nikolaou, Esther L. German, Mark Holloway, Beatriz F. Carniel, Mei Ling J. N. Chu, Kayleigh Arp, Elisabeth A. M. Sanders, Daniela M. Ferreira, Debby Bogaert

**Affiliations:** 1 Department of Paediatric Immunology and Infectious Diseases, Wilhelmina Children’s Hospital/University Medical Center Utrecht, Lundlaan 6, Utrecht, 3584 EA The Netherlands; 2 Department of Medical Microbiology, University Medical Center Utrecht, Heidelberglaan 100, Utrecht, 3584 CX The Netherlands; 3 Medical Research Council/University of Edinburgh Centre for Inflammation Research, Queen’s Medical Research Institute, University of Edinburgh, 47 Little France Crescent, Edinburgh, EH16 4TJ United Kingdom; 4 Department of Clinical Sciences, Liverpool School of Tropical Medicine, Pembroke Place, Liverpool, L3 5QA United Kingdom

**Keywords:** Microbiome, Pathogens, Influenza virus

## Abstract

*Streptococcus pneumoniae* is the main bacterial pathogen involved in pneumonia. Pneumococcal acquisition and colonization density is probably affected by viral co-infections, the local microbiome composition and mucosal immunity. Here, we report the interactions between live-attenuated influenza vaccine (LAIV), successive pneumococcal challenge, and the healthy adult nasal microbiota and mucosal immunity using an experimental human challenge model. Nasal microbiota profiles at baseline are associated with consecutive pneumococcal carriage outcome (non-carrier, low-dense and high-dense pneumococcal carriage), independent of LAIV co-administration. *Corynebacterium*/*Dolosigranulum*-dominated profiles are associated with low-density colonization. Lowest rates of natural viral co-infection at baseline and post-LAIV influenza replication are detected in the low-density carriers. Also, we detected the fewest microbiota perturbations and mucosal cytokine responses in the low-density carriers compared to non-carriers or high-density carriers. These results indicate that the complete respiratory ecosystem affects pneumococcal behaviour following challenge, with low-density carriage representing the most stable ecological state.

## Abstract (web summary)

Using a *Streptococcus pneumoniae* human challenge model, the authors here show that baseline nasal microbiota profiles are associated with pneumococcal acquisition and density, independent of live-attenuated influenza vaccine (LAIV) administration, and with mucosal cytokine responses to LAIV.

## Introduction

Respiratory tract infections (RTIs), such as pneumonia, are a major global health problem, accounting for ~15% of childhood mortality^1^. These infections are caused by bacteria such as *Streptococcus pneumoniae* that commonly reside in the healthy upper respiratory tract, where they are embedded in a complex microbial ecosystem, referred to as the microbiome. Carriage of these so-called pathobionts is a prerequisite for disease to develop^2^, with carriage density being associated with invasive pneumococcal pneumonia^3^, although factors governing acquisition and carriage—processes vital in RTI pathogenesis—are incompletely understood.

Both initial colonization and subsequent dynamics are likely to be impacted by the local microbiota through a process called colonization resistance, which can be direct or immune mediated^4,5^. This concept of colonization resistance may likely also prevent pathogen overgrowth in the respiratory tract^6,7^, and may therefore have a causal role on pneumococcal colonization and elimination.

The process of pneumococcal colonization is further affected by viral co-infections. Notably, during the 1918 Spanish influenza epidemic, the majority of influenza fatalities was likely caused by secondary pneumococcal pneumonia^8^. The role of influenza in acquisition and blooming of pneumococci is demonstrated in a controlled experimental infection model using wild-type influenza virus^9^, and in studies testing live-attenuated influenza vaccine (LAIV) in mice^10^ and humans^11,12^. Mechanistic pathways have been partly delineated^11,12^; however, less is known on the effects of viral infection on the resident respiratory microbiota and vice versa^13,14^.

To address these questions, we study the bacterial community dynamics following pneumococcal challenge and colonization, with and without antecedent viral infection using an experimental human pneumococcal challenge (EHPC) model. Healthy adult volunteers are challenged with a serotype 6B pneumococcal strain and randomized to receive either tetravalent inactivated influenza vaccine (control) or LAIV. In this model, a colonization rate of 50% is expected. For the primary study^15,16^, we found that antecedent LAIV vaccination, that is, LAIV administration followed by pneumococcal colonization, did not impact overall pneumococcal acquisition, yet did drive time to acquisition, and transiently increased pneumococcal carriage rate and density (approximately 10-fold) compared to controls.

We hypothesize that the nasal microbiota at baseline affects the likelihood and density of successful pneumococcal colonization. Furthermore, we hypothesize that the effect of pneumococcal challenge alone (i.e. without subsequent successful colonization) has limited effects on nasal microbiota dynamics and stability. Last, we anticipate that any perturbations caused by pneumococcal carriage are augmented in the group who received LAIV (i.e. during co-infections with attenuated influenza virus).

## Results

### Characteristics of the study population

**Fig. 1:**
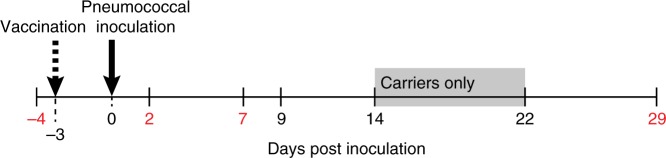
Schematic overview of the study design. Volunteers were screened for pneumococcal carriage on day −4, after which they received live-attenuated influenza vaccine (LAIV) or control vaccine on day −3 (dashed arrow), followed by pneumococcal inoculation on day 0 (solid arrow) and sampling visits on days 2, 7, 9 and 29. Only participants in whom *S. pneumoniae* 6B was detected by conventional culture at days 2, 7, and/or 9 were visited for sampling on days 14 and 21 (grey shaded area). Red numbers mark the time points at which microbiota data were available

Baseline characteristics of the participants are described in Supplementary Table 1. All volunteers were screened for pneumococcal carriage at baseline (Fig. 1); individuals positive for natural pneumococcal carriage were excluded from further analysis (*n* = 4; 3%). We analysed all 451 samples of 117 participants in total by 16S-based sequencing and *lytA*-quantitative PCR (qPCR) (Supplementary Table 2). Additionally, 115/116 baseline samples were screened for viral co-infection. As more extensively described in a separate manuscript^15,16^, 49 (41.9%) of the volunteers became high-dense and 27 (23.1%) became low-dense carriers. The remaining volunteers (*n* = 41 [35.0%]) were negative for pneumococcal carriage at either day 2, 7 or 9 (i.e. non-carriers). Carriage outcome was also tightly related to pneumococcal density of carriage as calculated by area under the log_10_-transformed (*lytA*) density–time curve (Supplementary Fig. 1).

### Baseline nasopharyngeal microbiota composition

**Fig. 2:**
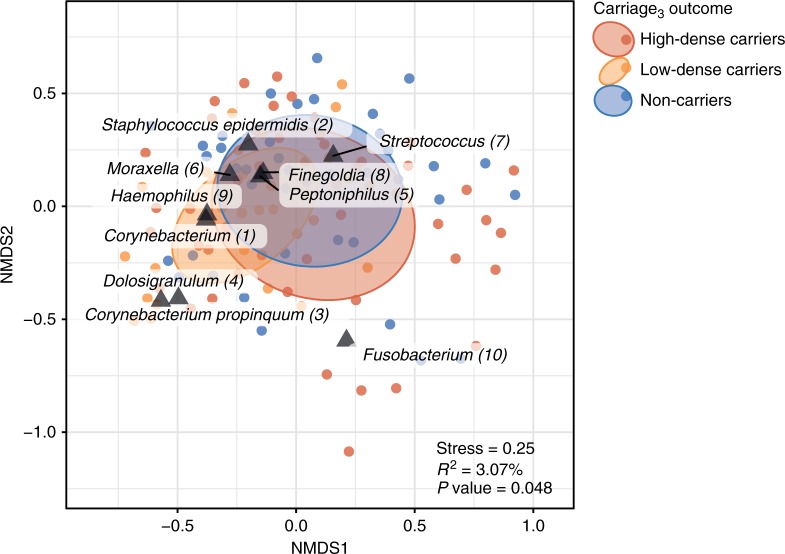
Baseline nasal microbiota composition in association with pneumococcal carriage_3_ outcome. Non-metric multidimensional scaling (NMDS) plot where each point represents the microbial community composition of one sample. Samples (*n* = 116) were coloured according to carriage_3_ outcome (red, high-dense carriers, *n* = 49; blue, non-carriers, *n* = 40 and orange, low-dense carriers, *n* = 27). The standard deviation of data points within carriage outcome groups is shown. In addition, the 10 highest ranked operational taxonomic units (OTUs) were simultaneously visualized (triangles). The stress value indicates how well the high-dimensional data are represented in the two-dimensional space; a value of ~0.2 indicates a reasonable representation. *P* values and effect sizes (*R*^2^) describing the strength and the significance of the association between baseline nasal microbiota and pneumococcal carriage outcome were generated using PERMANOVA tests, and are adjusted for the month, presence of any virus at baseline, the interactions between carriage outcome and the vaccination group/presence of any virus at baseline. See Table 1 for details

After sequencing and quality control, we observed a total of 343 operational taxonomic units (OTUs), representing 13 bacterial phyla. The presence of natural viral co-infection at baseline was detected in 9.6% of samples. We first assessed the associations between baseline nasal microbiota composition, natural viral co-infection (i.e. before pneumococcal inoculation and vaccination) and pneumococcal carriage outcome. We detected a significant association between baseline nasal microbiota composition and consecutive pneumococcal carriage outcome. This effect was stronger for carriage_3_ outcome, that is, when volunteers were stratified in high-dense, low-dense and non-carriers based on both conventional culture and qPCR data (permutational multivariate analysis of variance (PERMANOVA), *R*^2^ = 3.1%, *p* = 0.048; Fig. 2, Table 1 and Supplementary Fig. 2), compared to carriage_2_ outcome, where volunteers were dichotomized in carriers or non-carriers based on culturing only (Supplementary Fig. 3A and Supplementary Table 3A). The importance of semi-quantitative information on pneumococcal density in these analyses was further supported by a borderline significant association between baseline microbiota composition and density of pneumococcal colonization at day 2 (*R*^2^ = 1.7%, *p* = 0.076; Supplementary Table 3B). Interestingly, volunteers who became low-dense carriers demonstrated the lowest presence of natural respiratory viruses at baseline (before intervention) and showed the lowest rate of (replicating) influenza virus following LAIV vaccination compared to both high-dense and non-carriers (Supplementary Table 4). These associations were independent (Supplementary Table 4E). We thus studied the potential interaction between viral co-infection, microbiota composition and pneumococcal carriage_3_ outcome; we observed that the association between baseline microbiota and pneumococcal carriage acquisition was dependent on viral co-infection at baseline, but not on LAIV vaccination (*p* = 0.011 and *p* = 0.640, respectively; Table 1). Stratified analysis suggests, however, that the association between microbiota and carriage_3_ outcome was slightly weaker for the LAIV cohort compared to the control cohort (*R*^2^ = 2.9 vs. *R*^2^ = 4.2%, respectively; Supplementary Fig. 3B and 3C), suggesting interactions between both natural viral co-infection and iatrogenic LAIV infection, and (1) baseline nasal microbiota and (2) pneumococcal carriage receptiveness.

**Table 1:** Associations between baseline nasal microbiota and carriage_3_ outcome

Variable	Df	*F*	*R* ^2^	*P* value
Carriage_3_ outcome	2	1.79	3.07%	0.048
Month	4	0.88	3.02%	0.628
Any virus at baseline	1	0.74	0.64%	0.613
Carriage_3_ outcome: Vaccine	3	0.86	2.22%	0.640
Carriage_3_ outcome: Any virus at baseline	2	2.00	3.44%	0.011
Residuals	102		87.60%	
Total	114		100.00%	

The association between baseline nasal microbiota composition and carriage_3_ outcome was adjusted for the month of sampling (i.e. seasonal effects), the presence of any virus at baseline (day −4) and the interactions between carriage_3_ outcome and vaccine/presence of any virus at baseline (*n* = 116). These interactions (indicated by a colon) were included to properly assess the associations between baseline microbiota and carriage_3_ outcome, the latter of which could have been impacted by viral co-infection. Analyses were performed using PERMANOVA. See Supplementary Table 3 for the detailed assessment of associations between microbiota composition and carriage_2_ outcome/pneumococcal density

*PERMANOVA* permutational multivariate analysis of variance, *df* degrees of freedom, *F* *F* test statistic, *R*^2^ measure of variance

### Baseline microbiota clusters and pneumococcal challenge

**Fig. 3:**
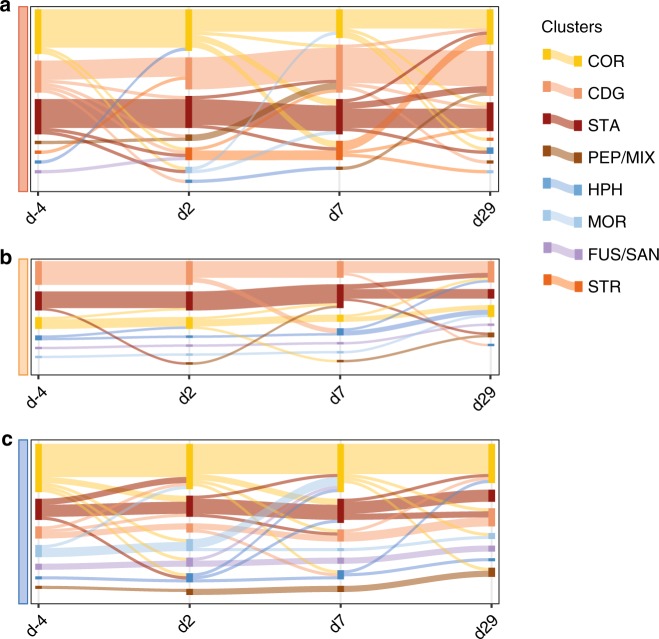
Dynamics of nasal microbiota profile membership. The number of samples in each cluster at each time point was visualized in alluvial diagrams, which were stratified by pneumococcal carriage_3_ outcome. Cluster membership was determined using average linkage hierarchical clustering based on the Bray–Curtis dissimilarity matrix. Clusters were characterized by *Staphylococcus* (*2*; STA); *Corynebacterium* *(3)* and *Dolosigranulum* (*4*; CDG); *Corynebacterium* (*1*; COR); *Haemophilus* (*9*; HPH), *Moraxella* (*6*; MOR), *Fusobacterium* (*10*; FUS), *Streptococcus* (*7*; STR), and *Peptoniphilus* *(5)*, *Finegoldia* *(8)*, *Anaerococcus* *(11)* and *Streptococcus salivarius* (*13*; PEP/MIX). The dynamics of nasal microbiota profile membership were shown for high-dense carriers (**a**), low-dense carriers (**b**) and non-carriers (**c**). The height of the figures corresponds with the total number of samples within that group. In addition, the height of the nodes and the thickness of the edges connecting the nodes is proportional to the number of samples. The number of samples in each cluster at each time point, stratified by carriage_3_ outcome, is provided in Supplementary Table 5. The number of cluster changes was lower in low-dense carriers compared to both high-dense and non-carriers (see Tables 2 and 3). A higher proportion of CDG profiles was observed in low-dense vs. non-carriers. Contrariwise, low-dense carriers specifically lacked STA-dominated profiles compared to both high-dense and non-carriers

To assess microbiota dynamics we performed an average linkage hierarchical clustering using Bray–Curtis dissimilarity. We found 18 clusters of microbiota profiles, of which 8 clusters comprised of ≥10 samples, representing in total 418/451 samples (Supplementary Fig. 4). We identified the OTUs that discriminated most between clusters using a random forest algorithm (referred to as biomarker species; Supplementary Fig. 5A). The largest cluster (STA; *n* = 132 [31.6%]) was characterized by *Staphylococcus* spp., followed by a cluster typified by several *Corynebacterium* (of which the most abundant was *Corynebacterium* *[3]*) and *Dolosigranulum* spp. (CDG; *n* = 104 [24.9%]), and a separate *Corynebacterium* (1) cluster (COR; *n* = 102 [24.4%]). The other five rarer clusters, characterized by *Haemophilus* spp. (HPH; *n* = 20 [4.8%]), *Peptoniphilus*, *Anaerococcus*, *Finegoldia* spp. and *Streptococcus salivarius* (PEP/MIX; *n* = 17 [4.1%]), *Moraxella* spp. (MOR; *n* = 17 [4.1%]), *Fusobacterium* (FUS; *n* = 14 [3.3%]) and *Streptococcus* spp. (STR; *n* = 12 [2.9%]), each comprised <5% of samples (Fig. 3, Supplementary Fig. 5B and Supplementary Table 5). In line with previous findings, the cluster distribution at baseline was significantly different between low-dense and non-carriers (Fisher’s exact test with Monte Carlo simulation; *p* = 0.039). Baseline differences were related to a higher proportion of CDG profiles in low-dense carriers vs. non-carriers (37.0 vs. 10.8%, respectively, *p* = 0.042). Contrariwise, although non-significant, low-dense carriers specifically lacked STA-dominated profiles compared to both non-carriers (18.5 vs. 43.2%, respectively, *p* = 0.058) and high-dense carriers (39.5%, *p* = 0.111). These results were confirmed by a stratified analysis where clustering was based on baseline samples only, ruling out potential confounding of these associations by profiles that emerge post-challenge.

### Baseline microbiota biomarkers and pneumococcal carriage

Using analysis of composition of microbiome (ANCOM), we identified OTUs present at baseline that were associated with pneumococcal carriage_3_ outcome, showing baseline *Corynebacterium (3)* and *Dolosigranulum (4)* were positively associated with low-dense carriers compared to non-carriers (Supplementary Fig. 6). metagenomeSeq analysis confirmed this association for *Corynebacterium* (*3*; Supplementary Fig. 7 and Supplementary Table 6). Moreover, using metagenomeSeq, we detected an additional consortium of lower abundant OTUs also related to carriage_3_ outcome. These OTUs were positively associated with both high-dense carriers compared to low-dense and non-carriers and belonged to the families *Prevotellaceae*, *Campylobacteraceae* and *Neisseriaceae*, which are (facultative) anaerobes, primarily residing in the oral cavity (Supplementary Fig. 7 and Supplementary Table 6). Lower abundance of these (facultative) anaerobes in low-dense carriers was also associated with a reduced microbial diversity compared to high-dense and non-carriers (Wilcoxon’s rank-sum test; *p* = 0.01 and *p* = 0.03, respectively; Supplementary Fig. 8A and Supplementary Table 7), and was independent of vaccination group (based on stratified analyses; Supplementary Fig. 8B and 8C and Supplementary Table 7).

### Mucosal cytokines, baseline microbiota and *S. pneumoniae*

**Fig. 4:**
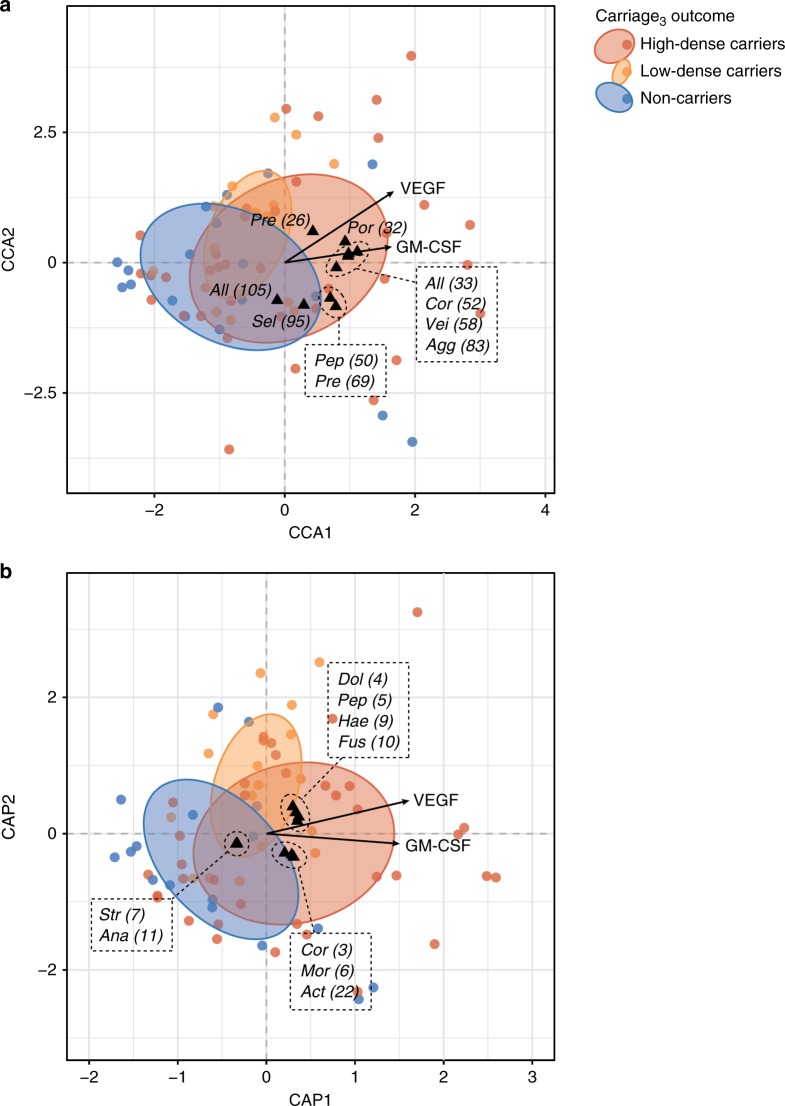
Associations between cytokine levels and nasal microbiota at baseline. Using canonical correspondence analysis (CCA) (**a**) and distance-based redundancy analysis (dbRDA) (**b**), we assessed the links between cytokine levels day 0 and baseline nasal microbiota. It is assumed that the dependent variables (log_10_ + 1-transformed relative abundance operational taxonomic units [OTUs]) respond in a unimodal or linear fashion to the predictor variables (log_2_-transformed cytokine levels) for CCA and dbRDA, respectively. We simultaneously plotted the samples (data points, *n* = 71), significant (*p* < 0.05) predictor variables (cytokines; arrows) and the OTUs that were most strongly associated with the first two axes (*n* = 10 for each axis, excluding overlapping OTUs). Samples were coloured according to carriage_3_ outcome (red, high-dense carriers, *n* = 36; blue, non-carriers, *n* = 18 and orange, low-dense carriers, *n* = 17), ellipses denote the standard deviation of the samples in each group. Note that carriage_3_ outcome was not accounted for when simultaneously modelling cytokine/microbiota data, yet still are clearly discriminated, suggesting that baseline microbiota and cytokine levels at day 0 (following live-attenuated influenza vaccine (LAIV) and prior to pneumococcal challenge) are related to pneumococcal receptiveness. Data separation by carriage_3_ outcome was higher when ordination was based on both microbiota and cytokine data (dbRDA and CCA; standardized absolute *β*-coefficient 0.43 and 0.32, respectively) compared to microbiota alone (non-metric multidimensional scaling [NMDS]; 0.22; Fig. 2). This was also true when coefficients were split between *X*- or *Y*-coordinates. An extensive description on our method to compare data separation by carriage_3_ outcome is provided in the Supplementary Methods section. CAP, constrained analysis of principal coordinates; GM-CSF, granulocyte–macrophage colony-stimulating factor; VEGF, vascular endothelial growth factor; *Cor*, *Corynebacterium*; *Dol*, *Dolosigranulum*; *Pep*, *Peptoniphilus*; *Mor*, *Moraxella*; *Fin*, *Finegoldia*; *Str*, *Streptococcus*; *Hae*, *Haemophilus*; *Fus*, *Fusobacterium*; *Ana*, *Anaerococcus*; *Act*, *Actinobacillus*; *Pre*, *Prevotella*; *All*, *Alloprevotella*; *Por*, *Porphyromonas*; *Vei*, *Veillonella*; *Agg*, *Aggregatibacter* and *Sel*, *Selomonas*. Numbers correspond with overall mean relative abundance rank

Mucosal cytokine data were assayed from samples collected at day 0, 2, 7, and 9 (Supplementary Fig. 9). We first assessed the links between baseline nasal microbiota and cytokine levels at day 0 (i.e. following LAIV vaccination), regardless of carriage_3_ outcome, using both canonical correspondence analysis (CCA) and distance-based redundancy analysis (dbRDA; Fig. 4). Intriguingly, the OTUs that were most strongly related to the first two axes of both CCA and dbRDA were mostly oral-type species, including *Streptococcus*, *Veillonella*, *Prevotella* and *Porphyromonas* spp., and other previously identified acquisition-associated microbiota members, including *Dolosigranulum* *(4)* and *Corynebacterium (3)* (dbRDA). Only granulocyte–macrophage colony-stimulating factor (GM-CSF) and vascular endothelial growth factor (VEGF) were consistently and significantly associated with baseline nasal microbiota (anova.cca-function; *p* < 0.05). Cytokine levels at day 0 were significantly lower in volunteers who became low-dense carriers compared to non-carriers, although this was only significant in the LAIV but not in the control group upon stratification (GM-CSF, interferon-α (IFN-α), interleukin-12 (IL-12), IL-17, IL-1β, IL-2 and IL-4, within LAIV; linear model, *p* < 0.05, Supplementary Table 8A). In general, volunteers who became high-dense carriers had intermediate cytokine levels at baseline. Following we combined baseline microbiota plus cytokine data to study whether the combined data improved the classification strength of carriage_3_ outcome. Indeed, combining these data showed larger data separation than microbiota data alone (Fig. 4), suggesting that both baseline microbiota composition and baseline host responses are driving pneumococcal receptiveness.

### Microbiata changes upon pneumococcal and LAIV challenge

We next analysed microbial community behaviour following pneumococcal challenge. We found that the overall microbiota composition following pneumococcal challenge became most profoundly different between high-dense carriers, non-carriers and low-dense carriers, at days 2 and 7 (PERMANOVA, *R*^2^ = 4.3%, *p* = 0.004 at day 2 and *R*^2^ = 4.7%, *p* = 0.002 at day 7), after adjustment for LAIV vaccination, the presence of virus at baseline and the interactions between carriage_3_ outcome and vaccine/natural virus at baseline. Results were identical after exclusion of the OTU corresponding with *S. pneumoniae* from the OTU table, suggesting a broader ecological impact of pneumococcal acquisition and challenge on microbiota perturbations than colonization of a single species (pneumococcus) alone. At day 29, when pneumococcal carriage was nearly eliminated, the microbiota composition between outcome groups became similar again (*R*^2^ = 1.8%, *p* = 0.505).

The strength of the impact of LAIV per se or the presence of replicating influenza virus at day 0/2 on the nasal microbiota composition was highest at day 2 (*R*^2^ = 1.7%) and diminished over time (*R*^2^ = 1.3% at both days 7 and 29). The differences in overall microbiota composition after baseline were paralleled by differences in *α*-diversity, unexpectedly with an increase in microbial diversity in non-carriers compared to high-dense carriers on days 2 and 7 (Supplementary Fig. 8 and Supplementary Table 7), suggesting ecological perturbations upon pneumococcal challenge independent of whether the strain was acquired.

### Microbiota dynamics following pneumococcal challenge

The previously described baseline differences in microbiota composition that were related to carriage_3_ outcome translated into variation in microbiota dynamics following pneumococcal challenge (Fig. 3). We measured microbiota profile shifts following pneumococcal challenge (i.e. at day 2) compared to baseline, and found less profile changes in low-dense carriers (11.1%), compared to high-dense carriers (28.2%, generalized linear models, *p* = 0.11), as well as non-carriers (31.4%, *p* = 0.07, Tables 2 and 3). In line, change in Bray–Curtis dissimilarity between baseline and day 2 was significantly larger in high-dense carriers compared to low-dense carriers (Table 3; *p* = 0.05). The large number of profile changes over the challenge interval in non-carriers underscores that pneumococcal exposure alone, without subsequent colonization or antecedent vaccination, may also perturb the ecological equilibrium.

**Table 2:** Microbiota cluster changes

Carriage_3_ outcome	Change	Total	%
High-dense carriers	11	39	28.2
Low-dense carriers	3	27	11.1
Non-carriers	11	35	31.4

The number of change transitions (Change; i.e. transition to another cluster) and the total number of transitions (Total; i.e. transition to the same or another cluster) over the challenge interval (i.e. days −4 to 2) are shown. The percentage (%) is the number of change transitions divided by the total number of transitions within carriage_3_ outcome groups

**Table 3:** Dynamics of microbiota composition associated with pneumococcal exposure

Outcome parameter	Comparison	Estimate	OR (95% CI)	*P* value
Microbiota cluster change^a^	High- vs. low-dense carriers	1.15	3.14 (0.78–12.59)	0.11
	Non- vs. low-dense carriers	1.3	3.67 (0.91–14.81)	0.07
	High-dense vs. non-carriers	−0.15	0.86 (0.32–2.32)	0.76
Bray–Curtis dissimilarity^b^	High- vs. low-dense carriers	0.11	1.11 (1.00–1.24)	0.05
	Non- vs. low-dense carriers	0.07	1.07 (0.96–1.2)	0.21
	High-dense vs. non-carriers	0.04	1.04 (0.94–1.14)	0.44

Differences in the number of change transitions^a^ (see Table 2) and Bray–Curtis dissimilarity^b^ over the challenge interval (i.e. days −4 to day 2) between carriage_3_ outcome groups were calculated. Results from generalized linear models^a^ and linear models^b^, including the β-coefficient (Estimate), odds ratio (OR) with the 95% confidence interval (CI) and the *p* value, are shown. These results support the trend of more stable microbial ecosystems following pneumococcal challenge in the low-dense carriage group compared to the other groups

### Cytokine responses following pneumococcal acquisition

Cytokine levels following LAIV and preceding pneumococcal challenge (i.e. at day 0) were lower in low-dense carriers compared to non-carriers (Supplementary Table 8A), which translated into lower cytokine levels over the whole study period (area under the curve analyses days 0 to 9; IFN-α, IL-12, IL-17, IL-2 and IL-4; linear model, *p* < 0.05, within LAIV; Supplementary Table 8B). In addition, non-carriers had higher levels of IL-1β and IFN-α before as well as following pneumococcal challenge when compared to high-dense carriers (*p* < 0.1, within LAIV; Supplementary Table 8B), suggesting that pneumococcal acquisition is related to variation in and extend of LAIV-induced mucosal inflammation.

## Discussion

We studied the association between the nasal microbial ecosystem and pneumococcal acquisition and density in the context of natural or induced (LAIV) viral co-infection in a controlled human co-infection model. Baseline nasal microbiota composition was associated with pneumococcal carriage_3_ outcome. Contrary to our hypothesis, the largest ecological differences (composition and mucosal inflammation at baseline and microbiota profile changes directly following pneumococcal exposure) were not observed between high-dense and non-carriers, but between low-dense and either high-dense/non-carriers. Furthermore, low-density carriage is associated with low cytokine levels and limited viral co-infections at baseline, and followed by the lowest LAIV replication and microbiota perturbations and least mucosal inflammation upon acquisition of *S. pneumoniae* compared to both high-dense and non-carriers. Finally, pneumococcal exposure also causes perturbations of nasal microbiota and host responses even where carriage is not established.

Evidence regarding the impact of nasal microbiota on an individual’s receptiveness of pneumococcal carriage as well as the ecological resonations following pneumococcal exposure is limited. Previous studies have focussed on the interactions between different respiratory pathogens only, largely disregarding the ecological background these bacteria are embedded in^17^. More recently, next-generation sequencing-based studies have emerged, describing associations between pneumococcal presence and density and the local microbiota^18–20^. For example, it has repeatedly been demonstrated that *Corynebacterium* and *Dolosigranulum* spp. abundances in infants are negatively associated with *S. pneumoniae* colonization in infants^18,19^. It remained however an open question whether the observed associations are causally linked. Disentangling cause–effect relationships between blooming of pathobionts, changes in host immune response and shifts in microbiota composition is complex, especially in cohorts which study respiratory infection, and require longitudinal and experimental study approaches like we used here.

Our current findings hint towards the existence of specific microbiota constellations, which control pneumococcal carriage receptiveness. Interestingly, the impact of nasal microbiota on pneumococcal carriage outcome was larger in controls than in LAIV recipients, suggesting interference of the normal ecological processes by influenza virus.

Paradoxically, we observed that important nasopharyngeal bacterial community members *Corynebacterium* and *Dolosigranulum* spp., which previously have been associated with infant respiratory health^7,21^, were lowest in abundance in non-carriers and highest in low-dense carriers followed by high-dense carriers at baseline, although the latter is relative, as pneumococcal abundance was rarely dominating the microbial community in our adult setting. In line, a recent community-based observation showed that co-colonization of *Streptococcus* and *Dolosigranulium* spp. was related to low abundance of *Streptococcus* spp^22^. In addition, we identified a species-rich consortium of low-abundant (facultative) anaerobic OTUs belonging to, among others, the *Prevotellaceae* and *Veillonellaceae* families, which were enriched in high-dense carriers and to a lesser degree non-carriers, but not present in low-dense carriers. These species appeared also strongly related to mucosal cytokine profiles. In previous studies, (early) presence of these bacteria in the nasopharynx in children has been associated with lack of microbiota stability over time^21^, premature microbiota maturation and an increased risk of consecutive respiratory infections^7^, as well as more severe disease at times of a RTI^23^. Furthermore, vaccination with pneumococcal conjugate vaccine-7 has been related to temporary enrichment of, among others, *Veillonella*, *Prevotella*, *Fusobacterium* and *Leptotrichia* spp. and non-pneumococcal streptococci^24^, suggesting the existence of a biological interaction with pneumococcus.

We therefore hypothesize that controlled low-dense colonization of *S. pneumoniae* might be the most beneficial phenotype for both host and microbe, based on less ecological perturbations and a reduced cytokine levels observed in low-dense carriers. In contrast, especially in individuals who failed to become colonized, we observed strong mucosal responses combined with increased microbiota profile changes following challenge were observed. This finding was in line with previous observations by our group, demonstrating that pneumococcal exposure not followed by colonization was still associated with augmented anti-protein immunoglobulin responses, suggesting induction of host immune pathways^25^. Apart from a difference in *Dolosigranulum* and *Corynebacterium* spp. in non-carriers compared to high-dense carriers, these phenotypes were very alike, which suggests that in both cases the physiological balance between microbiota and immune surveillance is perturbed, presumably contributing to a self-enforcing dysbiosis-inflammation cycle^26^. This feedback loop acknowledges the bidirectionality of the links between immune response and microbiota, which likely explains our observations in both high-dense carriers and non-carriers. The unimodal, rather than linear relationship between microbiota composition and carriage receptiveness has not been identified before, since we previously had no access to samples obtained before pneumococcal inoculation. In line with previous work from our group focussing on the relationship between LAIV and pneumococcal carriage^15,16^, our current findings underline the importance of molecular pneumococcal detection methods in discerning the low-dense carrier group.

Characterization of microbiota changes related to LAIV/influenza virus was challenging, given the relatively large pneumococcus-induced ecological perturbations observed in both volunteers who became high-dense and non-carriers. Our data suggest that LAIV only modestly modulates microbiota-mediated receptiveness, and in addition, impacts pneumococcal acquisition and density by the induced mucosal inflammation^13,14^.

Our study did not include a group without intervention, and we did not expect that pneumococcal exposure not followed by colonization would have such a strong effect on local microbiota in the absence of colonization. We anticipate that our current findings in healthy adults are nuanced when compared to risk populations for colonization and infection such as children and elderly, as both their microbiota composition differs, and their immune system is either immature or senescent, potentially allowing for the identification of a more pronounced impact of microbiota-driven colonization resistance^27^.

Future research should certainly take the impact of pathobiont exposure not followed by colonization into account, especially since exposure without acquisition might vary strongly between populations and groups and is not detected in surveillance studies. Furthermore, the experimental human challenge model may be used to explore interactions between pathobionts colonization and administration of pre-/probiotics.

We here showed that baseline nasal microbiota composition is relevant in determining the receptiveness to pneumococcal colonization in the context of antecedent LAIV administration. The use of molecular techniques to determine pneumococcal presence enabled us to detect new biological phenomena, showing that particularly low-dense pneumococcal carriage represents characteristics of a more stable mucosal microbiome–host equilibrium compared with either high-dense and non-carriers.

## Methods

### Ethics statement

Ethical approval was granted by the Liverpool East NHS Research Committee (14-NW-1460) and all participants gave written informed consent.

### Study design and participants

Details on the study design, in-/exclusion criteria and participants were previously published^15,16^ and can be found in the Supplementary Methods.

In brief, healthy non-smoking adults, aged 18–50 years, were enrolled in a single-centre, double-blinded, placebo-controlled trial (2015–2016). LAIV was administered prior to experimental inoculation with pneumococcus and pneumococcal colonization rate and density were determined^15,16^. Participants randomly received either LAIV and intramuscular placebo or intramuscular vaccination paired with nasal placebo. Pneumococcal inoculation was performed as previously described^28,29^. We excluded individuals who carried pneumococcus based on culture at baseline (i.e. day −4).

### Sample collection and pneumococcal detection

Nasal wash samples for pneumococcal detection were collected on days 2, 7, 9 and 29. Additional nasal washes were performed at days 14 and 22 in volunteers who were carriage positive at day 2, 7 and/or 9. Next, nasal washes were processed as described previously^28,29^. As per the study protocol, pneumococcal detection was performed using (1) conventional culture^28–31^ and (2) qPCR targeting the pneumococcal *lytA* gene^32^. Nasal lining fluid samples (Nasosorption™, Hunt Developments) for Luminex analysis were collected and stored at −80 °C as previously described^33^.

### Sample selection and DNA isolation microbiota analyses

We selected baseline (day −4) and day 2, 7 and 29 nasal wash samples (four time points; Fig. 1) for microbiota analyses. Bacterial DNA was isolated from 200 µL resuspended nasal wash pellet (see Supplementary Methods) by bead beating in phenol^32^ and quantified using a qPCR with primers directed at the 16S-rRNA gene^21,34^. DNA was subsequently eluted in one aliquot of 50 μL elution buffer and stored at −20 °C until further analysis.

### 16S-rRNA sequencing

Amplicon libraries of the 16S-rRNA gene (V4 region) were generated, and sequencing was executed as previously described^7^. Amplicon pools were paired-end sequenced in seven runs using an Illumina MiSeq instrument (Illumina Inc., San Diego, CA, USA). Bioinformatic processing included trimming, error correction, assembly and 97%-identity binning of reads into OTUs. Following removal of chimeric reads, OTUs were taxonomically annotated using SILVA. Details on processing, quality control and removal of environmental and procedural contaminants are described in the Supplementary Methods. After abundance filtering, a rarefied dataset was generated and used for downstream analyses. *α*-Diversity measures were averaged over 100 rarefactions. *β*-Diversity was assessed using the Bray–Curtis dissimilarity metric.

### Viral qPCR

Nucleic acids for viral qPCR were extracted from one aliquot of 250 µL oropharyngeal swab and/or 80–120 µL Nasosorption sample using the Purelink^TM^ Viral RNA/DNA Mini Kit (Life Technologies Corporation, Carlsbad, CA, USA) according to the manufacturer’s instructions. We tested for a broad panel of respiratory viruses using primers, probes and PCR assay conditions specific for adenoviruses, parainfluenza virus 1–4^35^, human bocavirus^36^, human coronavirus OC43, NL63 and 229E^37,38^, respiratory syncytial virus (A and B)^39,40^, human metapneumovirus^41^, human rhinoviruses, enteroviruses and human influenza virus A^42^ and B^43^ (Supplementary Table 9).

### Luminex analysis of nasal lining fluid

Cytokines were eluted from stored Nasosorption filters using 100 µL of assay buffer (Thermo Fisher). Samples were centrifuged for 10 min at 16,000 × *g* to clear them prior to acquisition. Samples were acquired using a 30-plex magnetic human Luminex Cytokine Kit (Thermo Fisher) and analysed on a LX200 (Bio-Rad) with xPonent3.1 software (Luminex Corp) following the manufacturer’s instructions. A representative subset of 12 cytokines was selected for further analyses (based on co-clustering analyses and literature^44^). Samples were analysed in duplicates and samples with a coefficient of variation >25% were excluded.

### Variable definitions

In the manuscript describing the initial results of the LAIV-EHPC project, focussing on the effect of LAIV on pneumococcal carriage, results based on both pneumococcal detection methods (i.e. conventional culture and molecular) were presented, underscoring the importance of the increased sensitivity of molecular techniques^15,16^. For this manuscript, we therefore decided to test two carriage outcome variables on the basis of nasal washes from days 2, 7 and 9: (1) carriage_2_ outcome (based on pneumococcal detection using conventional culture only), carriers, with a culture-positive sample at any point and non-carriers, who were culture-negative at all times; and (2) carriage_3_ outcome (combination of pneumococcal detection using both conventional culture and molecular techniques), coded as high-dense carriers (culture-positive at any point), low-dense carriers (qPCR-positive and culture-negative) and non-carriers (qPCR*-* and culture-negative at every point). Initial explorative analyses demonstrated higher explanatory power of carriage_3_ outcome, that is, the variable incorporating qPCR results. We therefore decided to use this outcome variable throughout the rest of the manuscript instead of carriage_2_ outcome.

### Statistical analysis

All analyses were performed in the R version 3.3.0 within R studio version 0.99.902. We provided a detailed schematic on the research questions/associations explored and a data analysis flow chart depicting an overview of the methods used (Supplementary Fig. 10). Detailed information on our statistical analysis can be found in the Supplementary Methods.

In short, using PERMANOVA tests, we studied the associations between carriage outcome and the overall microbiota composition at baseline and each subsequent time point. In conjunction, we assessed the association between microbiota composition and month (i.e. seasonal effects), the presence of virus(es) at baseline and the interaction between LAIV or presence of viruses at baseline and carriage outcome. The relationships between microbiota composition and carriage outcome were visualized using NMDS plots. Differentially abundant OTUs at baseline associated with pneumococcal carriage_3_ outcome were detected using several statistical techniques, including (1) ANCOM^45^ and (2) metagenomeSeq^46^. Differences in *α*-diversity according to carriage_3_ outcome were tested using (1) Wilcoxon’s rank-sum tests and (2) linear mixed-effects models with carriage outcome, time point and the interaction between carriage outcome and time points as fixed effects and subject as random effect. We used the multcomp package to determine significant differences within specific contrasts. To assess the microbiota dynamics related to carriage_3_ outcome over time, we performed an unsupervised average linkage hierarchical clustering based on the Bray–Curtis dissimilarity matrix. The optimal number of clusters and biomarkers for each cluster were determined as previously described^7^. Comparisons of cytokine levels according to carriage_3_ outcome was performed using a linear model, including vaccine, carriage outcome and the interaction between vaccine and carriage outcome. We simultaneously assessed the associations between baseline nasal microbiota and day 0 cytokine levels using CCA and dbRDA.

### Reporting summary

Further information on research design is available in the Nature Research Reporting Summary linked to this article.

## Supplementary information

Supplementary Information

Reporting Summary

## Data Availability

16S-rRNA sequencing data from this study are available from NCBI under BioProject accession number PRJNA421976. All other data are available in the manuscript (and its Supplementary Information files) or from the corresponding author upon reasonable request.
