# Supplementary material for: Interaction between the nasal microbiota and S. pneumoniae in the context of live-attenuated influenza vaccine
Source: Nat Commun. 2019 Jul 5;10:2981. doi: 10.1038/s41467-019-10814-9 (PMC6611866; doi:10.1038/s41467-019-10814-9)
Supplement: Supplementary file 1 — Supplementary Information [file 41467_2019_10814_MOESM1_ESM.pdf]

SUPPLEMENTARY INFORMATION

de Steenhuijsen Piters *et al*. Interaction between the nasal microbiota and *S. pneumoniae* in the context of live-attenuated influenza vaccine.

This file includes:

Supplementary Methods

Supplementary Figures 1-10

Supplementary Tables 1-9

Supplementary references

SUPPLEMENTARY METHODS

Details on study design, in-/exclusion criteria, procedures and participant safety were published previously[1,2].

Study design and participants

We recruited a cohort of participants who were enrolled in a single-centre, double-blinded, placebo-controlled trial (October 2015 - March 2016). LAIV was administered prior to experimental inoculation with pneumococcus. The interval between pneumococcal inoculation and vaccination was 3 days (Figure 1). Participants were randomized to receive either LAIV and intramuscular placebo or intramuscular vaccination paired with nasal placebo. We enrolled healthy, non-smoking individuals 17-48 years of age.

Ethical approval was granted by the Liverpool East NHS Research Committee (14-NW-1460), and all participants gave written informed consent. The study protocol was pre-registered (EudraCT 2014-004634-26).

In-/exclusion criteria

Exclusion criteria were: administration of influenza or any pneumococcal vaccination, or clinically confirmed disease of either, in the preceding two years; close contact with individuals judged to be at high risk of invasive pneumococcal disease (i.e. children under 5 years of age, immunosuppressed patients and elderly); allergies to study medication; current febrile illness; use of antibiotics, or immune-modulating medication. All female participants were required to practice effective contraception, and to provide a negative pregnancy test.

Vaccination and pneumococcal inoculation procedures

Participants were allocated by a permuted-block algorithm (blocks of 10, 1:1). Allocations were held in individual sealed envelopes. Each participant received either 1) nasal LAIV (Fluenz Tetra,

AstraZeneca, UK and FluMist, MedImmune, Netherlands, were used interchangeably due to procurement shortages) paired with intramuscular placebo (0.5 mL normal saline), or 2) nasal placebo (0.2 mL normal saline) paired with intramuscular Quadrivalent Inactivated Influenza Vaccination (Fluarix Tetra, GlaxoSmithKline, UK). Vaccine and placebo were prepared by dispensing nurses who were independent of the study team; participants were blindfolded during vaccine administration. Nasopharyngeal inoculation was performed using a laboratory pipette by depositing 0.1 mL broth containing 80,000 colony-forming-units (CFU) of *Streptococcus pneumoniae* serotype 6B (strain BHN418) into each nostril. Bacterial stocks were batch prepared from mid-log broth culture stored at -80°C, and independently tested by Public Health England laboratories for antibiotic sensitivity (Etest, bioMérieux, UK) and purity. Participants were inoculated within 30 minutes of stock dilution, and quantitative culture was used to confirm the dose received.

Participant Monitoring and Safety

Complete physical examination of participants was performed at enrolment. Full blood count assessment was performed at screening. Adverse events were recorded throughout the study; symptoms were documented as adverse events only if the investigator considered them to be severe events. Participants reported their oral temperature daily for 7 days following inoculation. Use of standby-medication (amoxicillin) was recorded. Participants colonised with *S. pneumoniae* for either ultimate or penultimate clinic visits received a 3-day course of amoxicillin at the end of the study.

Nasal wash sampling

Nasal washes were performed at day 2, 7 ,9 and 29 as previously reported[3,4]. For experimentally colonised participants as determined by culturing in both studies, additional washes were performed at days 14 and 22. In short, 5 mL 0.9% saline was instilled into each naris, which was repeated twice (i.e. total 10 mL per naris). In case <10 mL was returned, up to 40 mL normal saline was used.

Nasal wash processing and culturing

Next, nasal washes were processed as described previously[3,5]. Briefly, samples were centrifuged for 10 minutes at 3,345 x g, after which the supernatant was removed and stored and the pellet was resuspended in 100 µL of skim milk tryptone glucose glycerol (STGG) medium. We then took aliquots of 20 µL and 10 µL for pneumococcal detection and serial dilution, respectively. The remaining suspended pellet was resuspended in 800 µL STGG, of which 200 µL was used for molecular detection of pneumococcus and 16S-rRNA-based MiSeq sequencing. Pneumococcal detection, quantification and serotyping was performed as described previously[3].

Bacterial DNA isolation

Bacterial DNA from 200 µL sample was isolated by bead-beating in phenol[6] and quantified using a qPCR with primers directed at the 16S-rRNA gene[7,8]. DNA was subsequently eluted in one aliquot of 50µl elution buffer and stored at -20°C until further analyses, which included *lytA* qPCR and 16S-rRNA MiSeq sequencing.

Nasal wash *lytA* qPCR

S. pneumoniae was additionally identified and quantified using a qPCR targeting the autolysin gene (*lytA*)[9] as previously described[10]. Pneumococcal density (in copies mL^{-1} nasal wash) was calculated for samples with a C_T value within the range of detection (based on a standard curve ranging from 1 ng μL^{-1} to 0.00001 ng μL^{-1} pneumococcal DNA). For samples with a C_T value between the lower limit of detection and 40, densities were imputed and samples with a C_T value >40 or undetermined were deemed negative. Both culture and *lytA* density data were calculated per mL nasal wash returned[1,2].

Viral qPCR

At each time point, an oropharyngeal swab was collected for viral detection. Nucleic acids were extracted from one aliquot of 250 µL oropharyngeal swab using the Purelink™ Viral RNA/DNA Mini Kit (Life Technologies Corporation, Carlsbad, CA 92008 USA) according the manufacturer's instructions. RNA was synthesized into cDNA using a MultiScribe reverse transcriptase kit and

random hexamers. Each 100 µL reaction mixture contained 40 µL of eluted RNA and 60 µL of a mixture of 10 µL of 10x RT buffer, 22 µL $MgCl_2$ (25 µM), 20 µL of dNTP mixture (2.5 µM each dNTP), 5 µL random hexamer (50 µM), 2 µL of RNase inhibitor (20U μL^{-1}) and 2.5 µL of Multiscribe reverse transcriptase (all from PE Applied Biosystems). Thermal cycling conditions were described previously[11]. The RNA/DNA/cDNA template was tested in a real-time PCR using primers, probes and PCR assay conditions specific for adenoviruses, parainfluenza virus 1–4[12], human bocavirus[13], human coronavirus OC43, NL63, and 229E[14,15], respiratory syncytial virus (A and B)[16,17], human metapneumovirus[18], human rhinoviruses, enteroviruses, and human influenza virus A[19] and B[20] (Supplementary Table 9). Initially, a multiplex PCR was carried out to detect parainfluenza virus 1 and 3, parainfluenza virus 2 and 4 or human coronavirus OC43, NL63, and 229E. If positive, a monoplex species-specific PCR was performed. All other viral PCRs were performed separately for each virus. For each PCR a total reaction volume of 12.5 µL, containing 5 µL template, 6.25 µL Taqman Fast virus 1-step master mix (influenza A and B and RSV) TaqMan 2X Universal PCR Master Mix (all other viruses; Applied Biosystems), 1.25 µL primer/probe mix. Amplification and detection was performed on a StepOnePlus real-time PCR unit (Life Technologies) under the thermal cycling conditions described by the manufacturer. Swabs collected at baseline were subjected to the complete viral panel. We additionally tested samples collected at day 2, 7 and 29 for influenzavirus in participants who received LAIV.

Nasal lining fluid: Luminex analysis and viral detection at d0/d2

Nasosorption samples were collected at each time point. Each sample was centrifuged for 10 min at 3,220xg to separate the nasal lining fluid from the filter. Filter and fluid were then stored separately. Then, cytokines were eluted from stored Nasosorption filters (Hunt developments) using 100 µL of assay buffer (ThermoFisher) by centrifugation for 10 min at 3,220xg. Samples were centrifuged for 10 min at 16,000xg to clear them prior to acquisition. Samples (~40 µL) were acquired using a 30-plex magnetic human Luminex cytokine kit (ThermoFisher) and analysed on a LX200 (Biorad) with xPonent3.1 software (Luminex Corp) following manufacturer's instructions. A representative subset of 12 cytokines were selected for further analyses. Redundant/co-clustering cytokines were excluded,

whilst considering previous findings from our group[21]. Samples were analyzed in duplicates and Nasosorption samples with a CV > 25% were excluded. Next, eluted sample (~60 µL) and raw nasal lining fluid were pooled (resulting in 80-120µL of sample) and subjected to a viral qPCR panel as described above.

16S-rRNA sequencing

We selected baseline (day -4), day 2, 7 and 29 nasal wash samples (4 time points; see Figure 1) for microbiota analyses. After bacterial DNA isolation, amplicon libraries of the 16S-rRNA gene (V4 region) were generated. Sequencing was executed as previously described[6,22]. PCR amplicon libraries were generated by amplification of the 16S ribosomal RNA gene using barcoded primers directed at the V4 hypervariable region, as previously described[22]. Primer pair 533F/806R was used for amplification. Amplicon pools from samples and controls were sequenced in seven runs using an Illuminia MiSeq instrument (Illumina Inc., San Diego, CA, USA), resulting in paired-end 250 nucleotide reads. We applied an adaptive, window-based trimming algorithm (Sickle, version 1.33)[23] using a quality threshold of Q20 (as opposed to Q30[22]) and a length threshold of 150 nucleotides to filter out low quality reads/nucleotides. We the number of sequence errors was further reduced by applying an error correction algorithm (BayesHammer, SPAdes genome assembler toolkit, version 3.5.0)[24]. Next, reads were assembled into contigs (PANDAseq, version 2.9)[25,26] and demultiplexed (Qiime version 1.9.1; split_libraries.py)[27]. We removed singleton sequences and identified chimeras using both *de novo* and reference chimera identification. After removal of chimeric sequences, VSEARCH abundance-based greedy clustering was used to pick OTUs at a 97% identity threshold[28]. OTUs were then annotated by the Naïve Bayesian RDP classifier (version 2.2)[29] with a classification confidence of 50% (default)[30] and annotations were based on the 97% identity SILVA 119 release reference database[31].

Statistical analysis

All analyses were performed in the R version 3.3.0 within R studio version 1.0.136. All figures were created using the *ggplot2* R-package and edited using Illustrator CC.

We also provided a detailed schematic on the research questions/associations explored and a data analysis flow chart depicting an overview of the methods used (Supplementary Figure 10).

Variable definitions

In the manuscript describing the initial results of the LAIV-EHPC project, focussing on the effect of LAIV on pneumococcal carriage, results based on both pneumococcal detection methods (i.e. conventional culture and molecular) were presented, underscoring the importance of the increased sensitivity of molecular techniques[1,2]. For this manuscript we therefore decided to test two carriage outcome variables on the basis of nasal washes from day 2, 7 and 9: 1) $carriage_2$ outcome (based on pneumococcal detection using conventional culture only), 'carriers', with a culture positive sample at any point and 'non-carriers', who were culture-negative at all times; and 2) $carriage_3$ outcome (combination of pneumococcal detection using both conventional culture and molecular techniques), coded as 'high-dense carriers' (culture-positive at any point), 'low-dense carriers' (qPCR-positive and culture-negative) and 'non-carriers' (qPCR- and culture-negative at every point). Initial explorative analyses demonstrated higher explanatory power of $carriage_3$ outcome, i.e. the variable incorporating qPCR results. We therefore decided to use this outcome variable throughout the rest of the manuscript instead of $carriage_2$ outcome.

Nasal wash sample selection

In conformity with the original study[1,2], we excluded samples from volunteers who were randomized to receive LAIV, but presumably did not receive the required dose due a systematic medication dispensing error. In addition, we excluded two samples in which we detected >3,500 reads. A total of 451 samples from 117 volunteers were included. In 101/117 (86.3%) volunteers, all 4 samples were available; in 15/117 (12.8%) volunteers, samples from 3 time points were available and in 1/117 (0.9%) volunteers, samples from 2 time points were available.

Data normalization and filtering

We generated an abundance-filtered dataset by including only those OTUs that were present at or above a confident level of detection (0.1% relative abundance) in at least two samples, retaining 485 OTUs (0.3% of reads excluded)[32]. We generated a rarefied OTU-table at a sequence depth of 3,500 reads, calculated the relative abundance of OTUs and used this table as input for downstream analyses. α-diversity measures were calculated for 100 rarefactions at a sequencing depth of 3,500 reads and averaged. Analysis of composition of microbiomes (ANCOM) was performed on raw, non-rarefied data[33]. We selected OTUs that were present in >25% of the samples (at at least one time point) with a mean relative abundance if present of 0.1%. This way, OTUs were selected for the comparison of baseline microbiota profiles between carriage$_3$ outcomes. For *metagenomeSeq*-analyses we used the same selection of OTUs, though normalisation was performed using cumulative sum scaling (CSS)[34]. β-diversity was assed using the Bray-Curtis dissimilarity metric (dissimilarities based on relative abundance of species) and the Jaccard index ('distances' based on presence/absence of species).

Quality control of 16S-rRNA gene amplicon sequencing

OTU-tables were corrected for environmental and procedural contaminants on the basis of environmental (*n*=5) and procedural/laboratory control samples (*n*=66) that were taken at the moment of nasal wash collection and bacterial DNA isolation, respectively. Within the procedural control samples, we identified and removed those OTUs with a relative abundance of >1% in >5% (*n*=4) samples[35], removing 2.5% of all reads. These 28 OTUs included, 25 OTUs with at least a genus level annotation, of which 72.0% were previously reported as contaminating genera by Salter *et al*[36].

Based on our environmental control samples, we identified a subset of potential contaminating environmental OTUs that were also known bacterial community members in the upper respiratory tract, notably several streptococcal species, precluding us from simply removing these OTUs from the dataset. Alternatively, we hypothesized and subsequently verified that these OTUs would demonstrate a strong negative association between ($\log_{10}$+1-transformed) raw read counts and ($\log_{10}$-transformed)

bacterial density (linear model, beta-coefficient <-0.1 and $p<0.05$), indicating a larger impact of contaminating species on low density samples. We next screened the OTU-table using these criteria (considering only OTUs present in >30% of samples with a mean read count of 10) in an unsupervised manner, which resulted in a total of 24 unique, potentially contaminating OTUs. Plotting read counts of these OTUs in time suggested the existence of a batch effect, displaying several time intervals associated with varying degrees of contamination. We therefore ran a non-parametric change point analysis (*changepoint.np* package) to identify the cut-off points of these time intervals (i.e. the moments in time in which a shift in raw read counts was observed, hereafter referred to as 'change points'). Change points that were identified across ≥5 OTUs and thus were likely related to a consistent contaminating batch-effect, were selected and the OTUs in which any of these change points were identified (*n*=11) were adjusted. We first removed the negative correlation between raw read counts and bacterial density (selecting the residuals from a linear model fitted for each interval and OTU). These residuals (with mean=0) were then shifted to a new mean (y-axis translation) which was based on the (log_{10}+1-transformed) read count mean within high density samples (upper quadrant), in which the amount of contamination was expected to be low/non-existent. This new baseline was adjusted for the total number of reads identified in each sample.

Furthermore, we included 7 mock communities, consisting of 12 bacterial species commonly observed in the upper respiratory tract (i.e. *Bacteroides fragilis*, *Haemophilus influenzae*, *Streptococcus pneumoniae*, *Streptococcus pyogenes*, *Klebsiella oxytoca*, *Klebsiella pneumoniae,* haemolytic *Streptococcus* group A*, Pseudomonias aeruginosa*, *Staphylococcus epidermidis*, *Staphylococcus aureus* and *Moraxella catarrhalis*). Equivalent amounts of DNA isolated from these species were combined and included as internal controls in each Illumina MiSeq run.

PERMANOVA and non-metric multidimensional scaling (nMDS)

Global microbiota differences between $carriage_{2/3}$ outcomes at baseline and at subsequent time points were visualised using non-metric multidimensional scaling (nMDS; *metaMDS* function in the *vegan* package; trymax=100)[37] based on the Bray-Curtis dissimilarity matrix. Ellipses representing the

standard deviation of data points were calculated using the internal *veganCovEllipse* function. Stress-values, which indicate how well the ordination captured the high-dimensional data (i.e. a measure of goodness-of-fit), were reported. We tested whether a nMDS-visualisation in a higher dimensional space would decrease the stress of the ordination using a scree plot (1-6 dimensions tested). Based on our findings (balancing number of dimensions, reduction in stress-value and interpretability of the plot) we decided to provide a three-dimensional nMDS plot as a supplementary figure (Supplementary Figure 2).

To assess the associations between nasal microbiota and pneumococcal carriage receptiveness and dynamics, while adjusting for potential variables of influence, we performed permutational multivariate analysis of variance (PERMANOVA)-tests (*adonis* function of the *vegan* package; Bray-Curtis dissimilarity, 999 permutations). We ran these tests at baseline and for each time point following pneumococcal inoculation. At baseline, we included $carriage_{2/3}$ outcome, month of sampling, the presence of any virus at baseline, the interaction between $carriage_{2/3}$ outcome and both vaccination group and the presence of any virus at baseline. These interactions were included because especially for LAIV, an effect on $carriage_{2/3}$ outcome may be expected, based on previous results[1,2]. However, since LAIV was allocated in a randomized manner and administered after baseline, this effect should not be tested for at this time point. At time points following pneumococcal inoculation we assessed the impact of $carriage_3$ outcome (i.e. pneumococcal exposure/colonization) on nasal microbiota, adjusting for vaccination group, presence of any virus at baseline, the interaction between $carriage_{2/3}$ outcome and both vaccination and the presence of any virus at baseline. We ran these tests both with and without the OTU representing *S. pneumoniae* (which was excluded before rarefactions) to quantify whether changes in overall microbiota composition were mainly driven by introduction of pneumococcus. P-values and R^2-values accompanying nMDS-plots were based on the PERMANOVA-tests described above.

α-diversity

We visualised and analyses three measures of α-diversity: the number of observed species, the Shannon index and the Simpson index. For each α-diversity measure, differences between $carriage_3$

outcomes at baseline were assessed using Wilcoxon rank sum tests and at each time point using mixed linear models. For the unstratified analyses, mixed linear models were fitted including the interaction between $carriage_3$ outcome and time point as fixed effects and subject as random effect. Post-hoc tests on contrasts of interest (i.e. differences between $carriage_3$ outcomes at each time point) were performed using the *multcomp* package[38]. We adjusted for multiple testing using the 'single-step' procedure (*multcomp* default), except when stated otherwise. To stratify our results for the effect of vaccine, we ran a similar mixed linear model, although this time including the interaction between $carriage_3$ outcome, time point and vaccination group as fixed effects and subject as random effect. Within vaccine groups, we again extracted the contrasts of interest (i.e. differences between $carriage_3$ outcomes at each time point).

Clustering

Especially since we observed a bimodal relative abundance distribution in some OTUs, we complemented our supervised analyses using *metagenomSeq* (see below) with a clustering approach. Individuals were clustered using unsupervised average linkage hierarchical clustering based on the Bray-Curtis dissimilarity matrix, as described before[22,39]. The number of clusters was determined based on the Silhouette and Calinski-Harabasz indices (*fpc* package)[40]. Clusters consisting ≥10 samples were considered for subsequent analyses. Subsequently, using a random forest algorithm, we identified the OTUs that discriminated most between clusters (Supplementary Figure 5), based on which cluster names were determined. Cluster membership in relation to 1) time point of sample collection, 2) vaccination group, 3) $carriage_3$ outcome and 4) the per-sample microbiota profile (stacked bar chart of the 15 most abundant OTUs) was depicted in a dendrogram. Furthermore, the proportion of samples within each cluster at each time point was visualised for each $carriage_3$ outcome using 1) stacked bar plots and 2) an alluvial diagram (*ggvisSankey*-function within the *googleVis* package)[41]. The stacked bar plots were used to visualise both the absolute number of samples that were binned to each cluster as well as the distribution of cluster memberships as a percentage of the total number of samples within each $carriage_3$ outcome. Associations between cluster distribution and $carriage_3$ outcome was assessed using Fisher's exact tests. A stratified analysis where clustering was

based on baseline samples only, was performed to rule out potential confounding of these associations by profiles that emerge post-challenge.

Microbiota change over time (focussing on the challenge interval spanning baseline to day 2) was assessed using 1) the number of cluster changes (e.g. from *Stapylococcus* [STA] to *Moraxella* [MOR]) vs the total number of cluster transitions per $carriage_3$ outcome and 2) the change in Bray-Curtis dissimilarity per $carriage_3$ outcome.

Detection of differentially abundant OTUs: metagenomeSeq and ANCOM

Both the *metagenomSeq*-package[34] and *ANCOM*-package[33] were used to determine differentially abundant OTUs between $carriage_3$ outcomes at baseline. Analyses were performed without accounting for vaccine, only including $carriage_3$ outcome as predictor. For *metagenomeSeq*, we additionally added a normalisation factor as predictor (default). For both packages, information on differentially abundant OTUs between $carriage_3$ groups (if applicable within vaccination groups) was extracted using contrast matrices.

Cytokine

Cytokine data were $\log_2$-transformed; missing values were imputed with the mean for that cytokine. Stratified analyses were performed using a linear model including $carriage_3$ outcome, vaccination group and the interaction between $carriage_3$ outcome and vaccination group as independent variables. Relevant contrasts were extracted using contrast matrices and the *multcomp*-package. We tested 1) cytokine levels at day 0 and 2) area-under-the-curves (AUCs; day 0 tot day 9) of cytokine levels. To study the relationship between nasal microbiota and the mucosal host immune response we used canonical correspondence analysis (CCA, *cca* function of the *vegan* package, 999 permutations) and distance-based redundancy analysis (dbRDA; *capscale* function of the *vegan* package, 999 permutations). For both functions, the $\log_{10}$+1-transformed rarefied OTU-table was used as outcome variable. Significant terms (i.e. cytokines) were determined using *anova.cca* (*vegan* package). Results of both CCA and dbRDA were visualised in a two-dimensional space (based on the first two axes), including samples (data points), significant terms (i.e. cytokines, arrows), bacterial species most

strongly associated with the first two axes (n=10 species with the highest absolute scores). Furthermore, $carriage_3$ outcomes were visualised using confidence ellipses (as previously described).

To compare how well the data were separated by $carriage_3$ outcome for ordination methods incorporating both microbiota and cytokine data, versus microbiota data alone, we regressed $carriage_3$ outcome against X- and Y-coordinates for each given sample, stratified by method used (i.e. nMDS [Figure 2], dbRDA and CCA [Figure 4]). Beta-coefficients for levels of $carriage_3$ outcome (i.e. high- or low-dense carriers and non-carriers) correspond with data separation in X- and Y-directions within each ordination space tested. Beta-coefficients were mean-centered and scaled for comparability across models. The average of the absolute values of these standardized beta-coefficients is a measure of data separation driven by pneumococcal carriage outcome.

SUPPLEMENTARY FIGURES

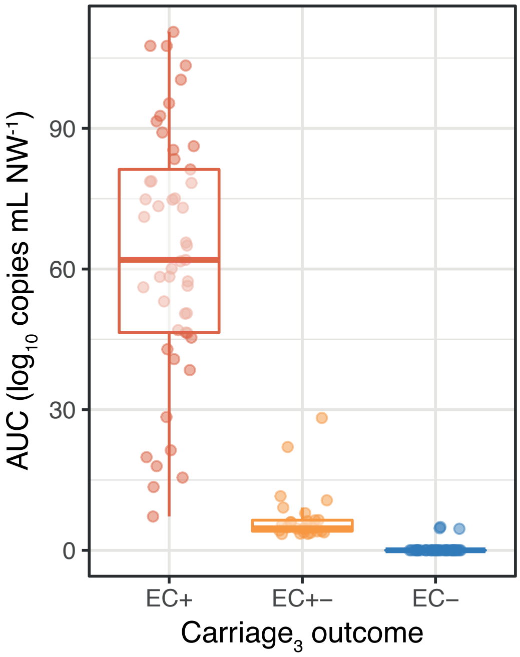

Supplementary Figure 1 - Relationship between carriage$_3$ outcome and pneumococcal density. The (categorical) carriage$_3$ outcome variable was compared to the (continuous) area under the log_{10}-transformed density-time curve variable, underlining the high correlation between these two variables (Wilcoxon rank sum test; $p<2.2 \times 10^{-16}$ and $p=1.2 \times 10^{-12}$ for high- vs low-dense carriers and low-dense vs non-carriers, respectively). Box plots represent the 25th and 75th percentiles (lower and upper boundaries boxes, respectively), the median (middle horizontal line), and measurements that fall within 1.5 times the interquartile range (IQR; distance between 25th and 75th percentiles; whiskers). Red, EC+, high-dense carriers, *n*=49; blue, EC-, non-carriers, *n*=41 and orange, EC+-, low-dense carriers, *n*=27).

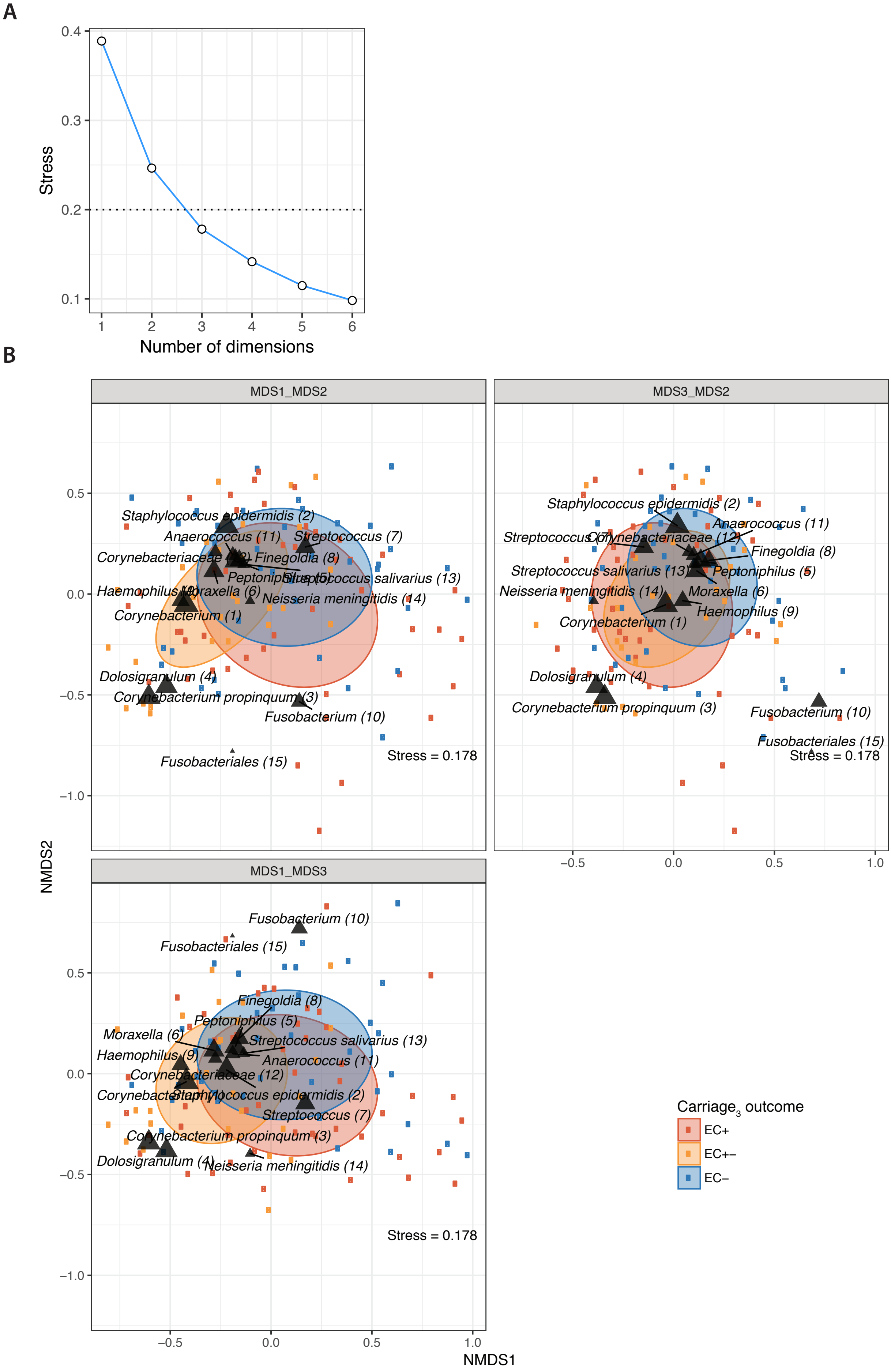
A
Stress
Number of dimensions
0.4
0.3
0.2
0.1
1
2
3
4
5
6
B
MDS1_MDS2
MDS3_MDS2
MDS1_MDS3
NMDS2
NMDS1
Staphylococcus epidermidis (2)
Anaerococcus (11)
Streptococcus (7)
Corynebacteriaceae (12)
Finegoldia (8)
Peptoniphilus (5)
Streptococcus salivarius (13)
Haemophilus (9)
Moraxella (6)
Neisseria meningitidis (14)
Corynebacterium (1)
Dolosigranulum (4)
Corynebacterium propinquum (3)
Fusobacterium (10)
Fusobacteriales (15)
Stress = 0.178
0.5
0.0
−0.5
−1.0
1.0
Carriage$_3$ outcome
EC+
EC+−
EC−

Supplementary Figure 2 – **Scree plot and three-dimensional nMDS of baseline nasal microbiota composition in relation to carriage$_3$ outcome. (A)** Scree plot to depict the relationship between the number of (nMDS)-dimensions and stress. Naturally, the stress will reduce by increasing the number of dimensions, however only a maximum number of three dimensions can reasonably be interpreted. When using three dimensions the stress-value drops well below 0.2[42], suggesting that the data is properly ordinated at this number of dimensions. **(B)** Three-dimensional nMDS plot. The main data structure visualized using the two-dimensional plot appears to be preserved when plotting the same data in three dimensions. Red, EC+, high-dense carriers, *n*=49; blue, EC-, non-carriers, *n*=40 and orange, EC+-, low-dense carriers, *n*=27).

A

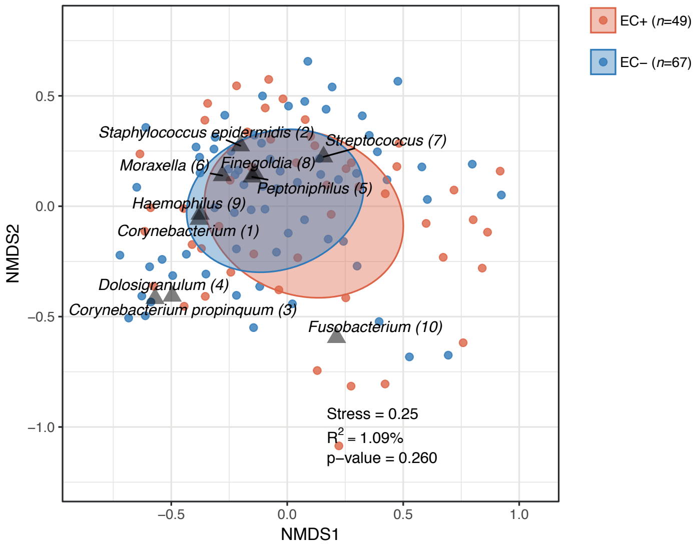

EC+ (n=49)
EC– (n=67)
Staphylococcus epidermidis (2)
Streptococcus (7)
Moraxella (6)
Finegoldia (8)
Peptoniphilus (5)
Haemophilus (9)
Corynebacterium (1)
Dolosigranulum (4)
Corynebacterium propinquum (3)
Fusobacterium (10)
Stress = 0.25
R2 = 1.09%
p–value = 0.260
NMDS1
NMDS2
0.5
0.0
−0.5
−1.0
1.0

B

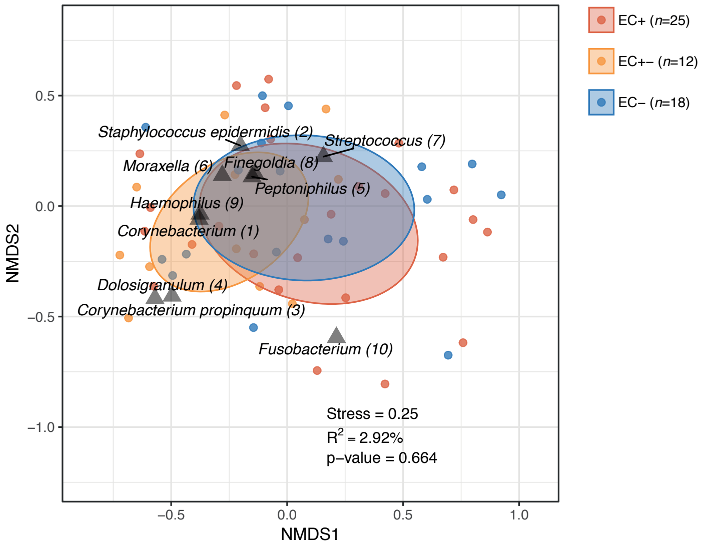

EC+ (n=25)
EC+– (n=12)
EC– (n=18)
Staphylococcus epidermidis (2)
Streptococcus (7)
Moraxella (6)
Finegoldia (8)
Peptoniphilus (5)
Haemophilus (9)
Corynebacterium (1)
Dolosigranulum (4)
Corynebacterium propinquum (3)
Fusobacterium (10)
Stress = 0.25
R2 = 2.92%
p–value = 0.664
NMDS1
NMDS2
0.5
0.0
−0.5
−1.0
1.0

C

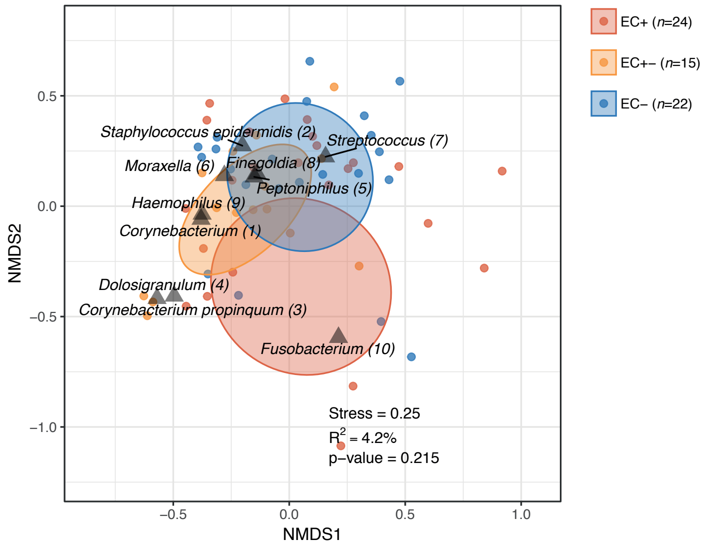

EC+ (n=24)
EC+– (n=15)
EC– (n=22)
Staphylococcus epidermidis (2)
Streptococcus (7)
Moraxella (6)
Finegoldia (8)
Peptoniphilus (5)
Haemophilus (9)
Corynebacterium (1)
Dolosigranulum (4)
Corynebacterium propinquum (3)
Fusobacterium (10)
Stress = 0.25
R2 = 4.2%
p–value = 0.215
NMDS1
NMDS2
0.5
0.0
−0.5
−1.0
1.0

Supplementary Figure 3 - Non-metric multidimensional scaling (nMDS) plots visualizing the baseline nasal microbiota composition in association with pneumococcal carriage$_{2/3}$ outcome, stratified by vaccine. The panels represent: carriage$_2$ outcome (*n*=116) **(A)**, carriage$_3$ outcome in LAIV (*n*=55) **(B)** and carriage$_3$ outcome in controls (*n*=61) **(C)**. Each point represents the microbial community composition of one sample. Samples were coloured according to carriage$_{2/3}$ outcome (red, EC+, [high-]carriers; blue, EC-, non-carriers and orange, EC+-, low-dense carriers). The standard deviation of data points within carriage outcome groups is shown. In addition, the 10 highest ranked OTUs were simultaneously visualized (triangles). The stress value indicates how well the high-dimensional data are represented in the two-dimensional space; a value of ~0.2 indicates a reasonable representation[42]. P-values and effect sizes (R^2) describing the strength and the significance of the association between baseline nasal microbiota and pneumococcal carriage outcome were generated using PERMANOVA-tests, and are adjusted for the month, presence of any virus at baseline, the interactions between carriage outcome and vaccination group (only for panel **A**)/presence of any virus at baseline. The strength of the association between baseline microbiota and carriage$_3$ outcome is weakened in individuals who received LAIV compared to controls, suggesting that LAIV may perturb colonization resistance conveyed by the microbiota. See Table 1 and Supplementary Table 3 for details.

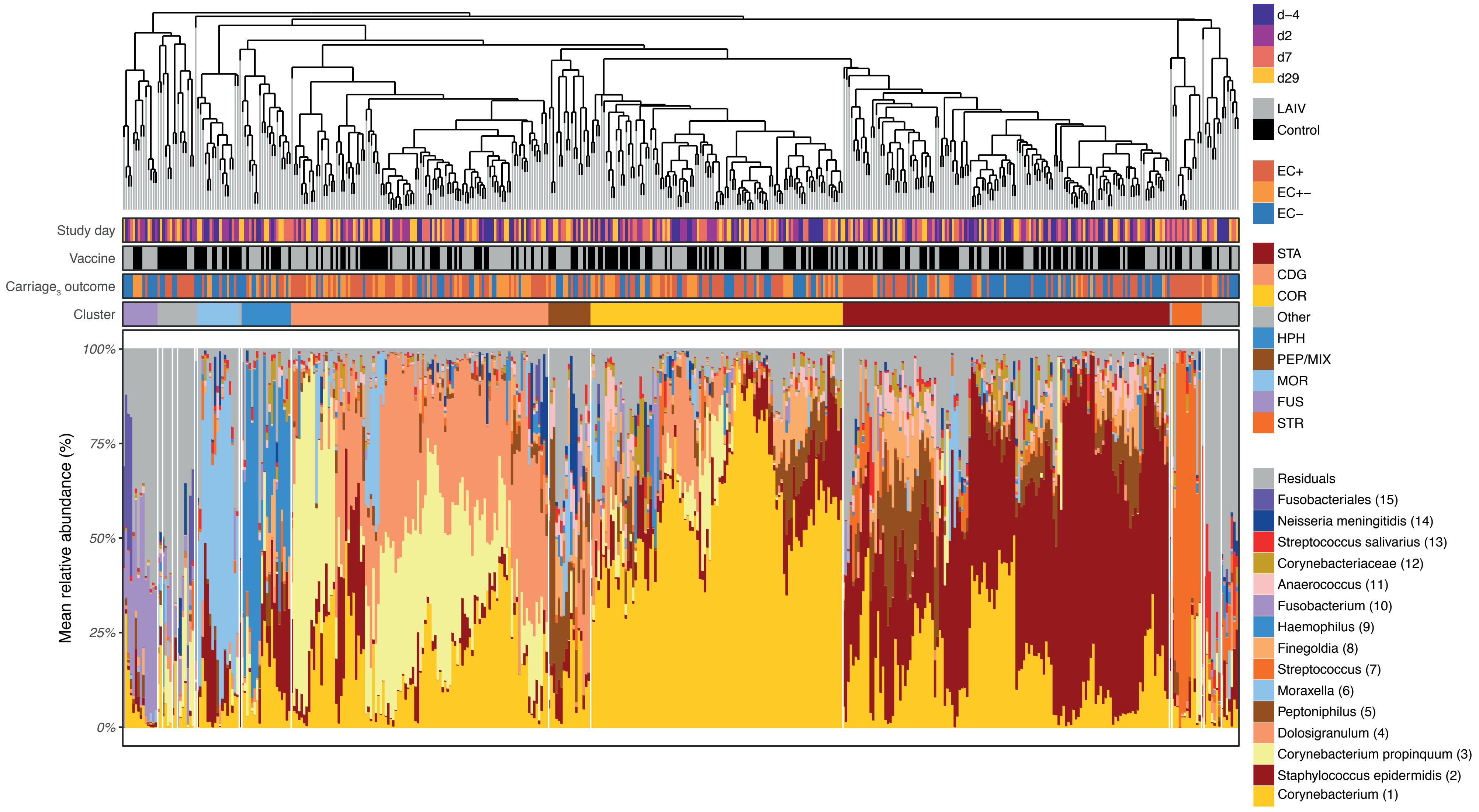

Study day
Vaccine
Carriage$_3$ outcome
Cluster
Mean relative abundance (%)
100%
75%
50%
25%
0%
d−4
d2
d7
d29
LAIV
Control
EC+
EC+−
EC−
STA
CDG
COR
Other
HPH
PEP/MIX
MOR
FUS
STR
Residuals
Fusobacteriales (15)
Neisseria meningitidis (14)
Streptococcus salivarius (13)
Corynebacteriaceae (12)
Anaerococcus (11)
Fusobacterium (10)
Haemophilus (9)
Finegoldia (8)
Streptococcus (7)
Moraxella (6)
Peptoniphilus (5)
Dolosigranulum (4)
Corynebacterium propinquum (3)
Staphylococcus epidermidis (2)
Corynebacterium (1)

Supplementary Figure 4 - Dendrogram visualizing an average linkage hierarchical clustering of samples based on the Bray–Curtis dissimilarity matrix. The length of the branches of the tree structure corresponds with the similarities between samples. Information on 1) time point of sample collection, 2) vaccination group, 3) $carriage_3$ outcome and 4) cluster membership is depicted adjacent to the branch ends in colour-coded horizontal panels. Stacked bar charts show the relative abundance of the 15 highest-ranked operational taxonomic units (OTUs) and of residual bacteria. OTUs are color coded according to phylum: Firmicutes, red; Proteobacteria, blue; Actinobacteria, yellow; Bacteroidetes, green and Fusobacteria, purple. On the basis of clustering indices, an optimal number of 18 clusters was identified, 8 of which comprised more than 10 study samples (*n*=418). Classifier taxa of these 10 clusters are depicted in Supplementary Figure 5A. Clusters were characterized by *Staphylococcus* (2) (STA); *Corynebacterium* (3) and *Dolosigranulum* (4) spp. (CDG); *Corynebacterium* (1; COR); *Haemophilus* (9; HPH), *Moraxella* (6; MOR), *Fusobacterium* (10; FUS), *Streptococcus* (7; STR), and *Peptoniphilus* (5), *Finegoldia* (8), *Anaerococcus* (11) and *Streptococcus salivarius* (13; PEP/MIX). Gray panels mark individuals not included in any of these 8 clusters. Repeated samples from individuals were included in this clustering analysis to optimize cluster identification and increase comparability across time points. Red, EC+, high-dense carriers; blue, EC-, non-carriers and orange, EC+-, low-dense carriers.

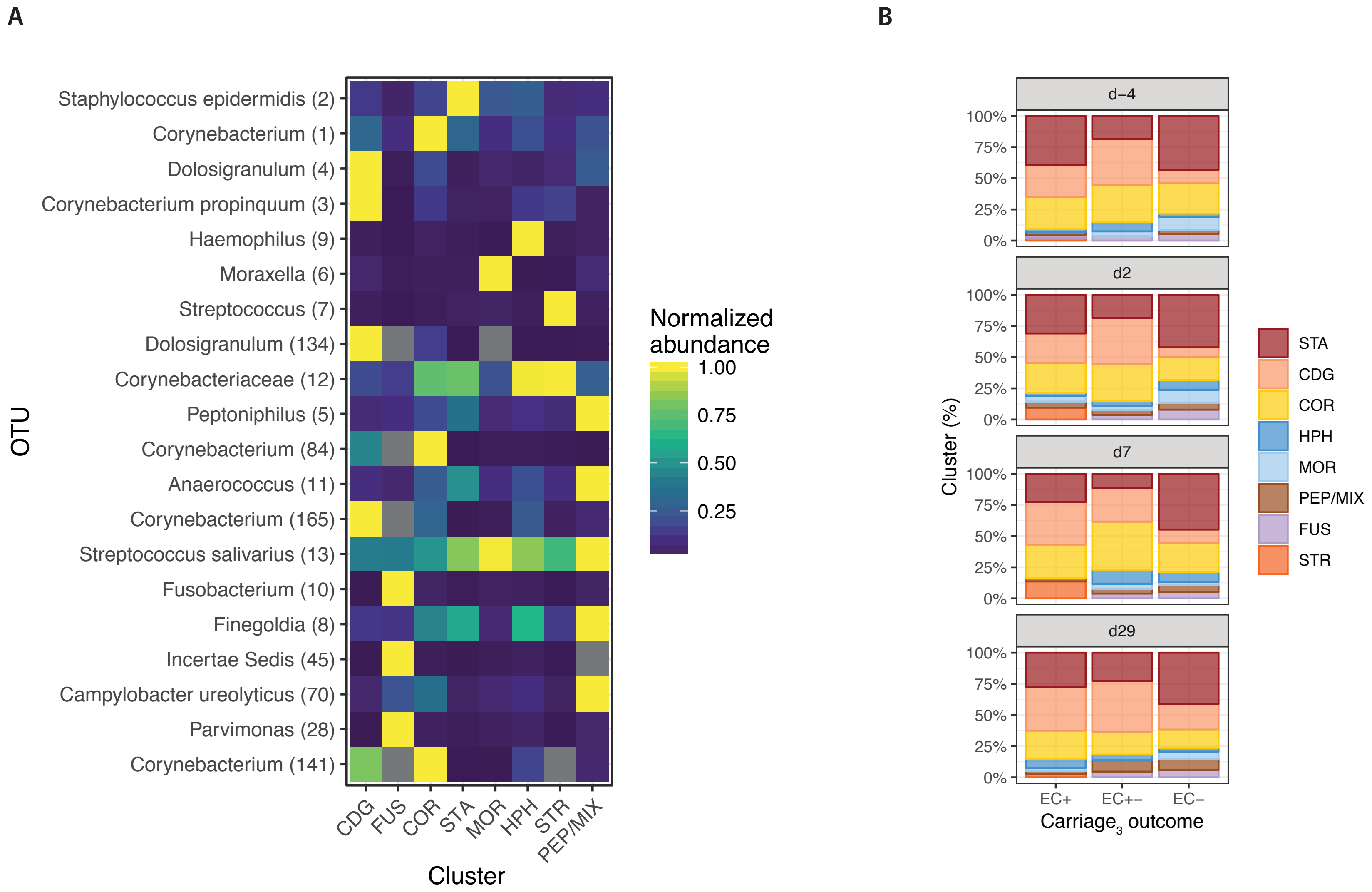
A
OTU
Staphylococcus epidermidis (2)
Corynebacterium (1)
Dolosigranulum (4)
Corynebacterium propinquum (3)
Haemophilus (9)
Moraxella (6)
Streptococcus (7)
Dolosigranulum (134)
Corynebacteriaceae (12)
Peptoniphilus (5)
Corynebacterium (84)
Anaerococcus (11)
Corynebacterium (165)
Streptococcus salivarius (13)
Fusobacterium (10)
Finegoldia (8)
Incertae Sedis (45)
Campylobacter ureolyticus (70)
Parvimonas (28)
Corynebacterium (141)
CDG
FUS
COR
STA
MOR
HPH
STR
PEP/MIX
Cluster
Normalized abundance
1.00
0.75
0.50
0.25
B
Cluster (%)
d−4
d2
d7
d29
100%
75%
50%
25%
0%
EC+
EC+−
EC−
Carriage3 outcome
STA
CDG
COR
HPH
MOR
PEP/MIX
FUS
STR

Supplementary Figure 5 – Random forest classifier taxa of the clusters and cluster distribution in time. (A) Heatmap of mean relative abundance of the 20 OTUs that most strongly discriminated between microbiota clusters. Cluster membership was determined using average linkage hierarchical clustering based on the Bray-Curtis dissimilarity matrix. Colours correspond with row wise normalized relative abundances (i.e. yellow indicates the maximum relative abundance of that OTU across clusters, deep purple indicates the minimum relative abundance). The cluster distribution at each time point is shown in panel **(B)**. Bars correspond with the relative cluster distribution (number of samples belonging to a given cluster out of the total number of samples, stratified by $carriage_3$ outcome). For cluster abbreviations see legend Supplementary Figure 4.

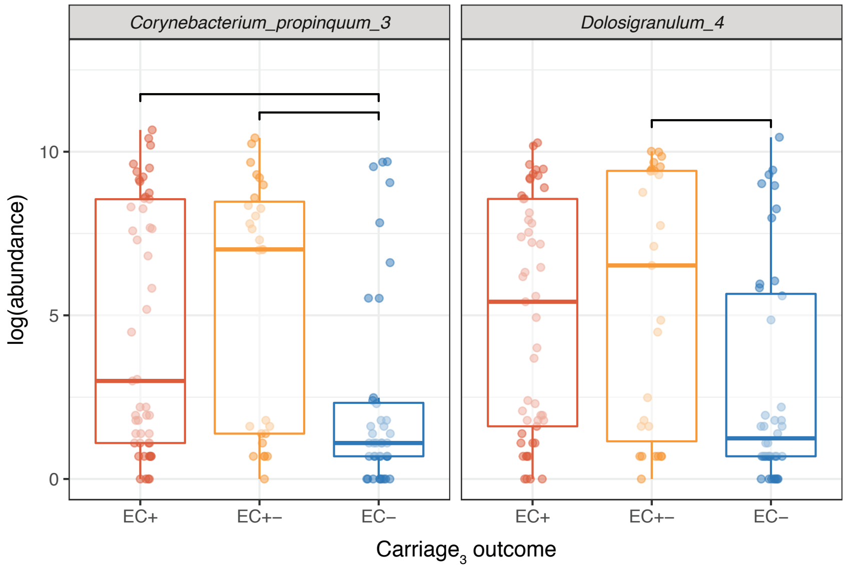

Supplementary Figure 6 – Differentially abundant OTUs at baseline between carriage$_3$ outcome phenotypes (ANCOM). Differentially abundant OTUs were detected using ANCOM based on a linear model including carriage outcome as dependent variable. Differentially abundant OTUs between high-dense carriers vs non-carriers, low-dense carriers vs non-carriers and low-dense carriers vs high-dense carriers were extracted. Box plots represent the 25th and 75th percentiles (lower and upper boundaries boxes, respectively), the median (middle horizontal line), and measurements that fall within 1.5 times the interquartile range (IQR; distance between 25th and 75th percentiles; whiskers). Significantly higher levels of *Corynebacterium propinquum (3)* and *Dolosigranulum (4)* were detected in low-dense carriers (and to a lesser extent high-dense carriers) compared to non-carriers. * indicates a significant difference after correction for multiple testing. Red, EC+, high-dense carriers, *n*=49; blue, EC-, non-carriers, *n*=40 and orange, EC+-, low-dense carriers, *n*=27).

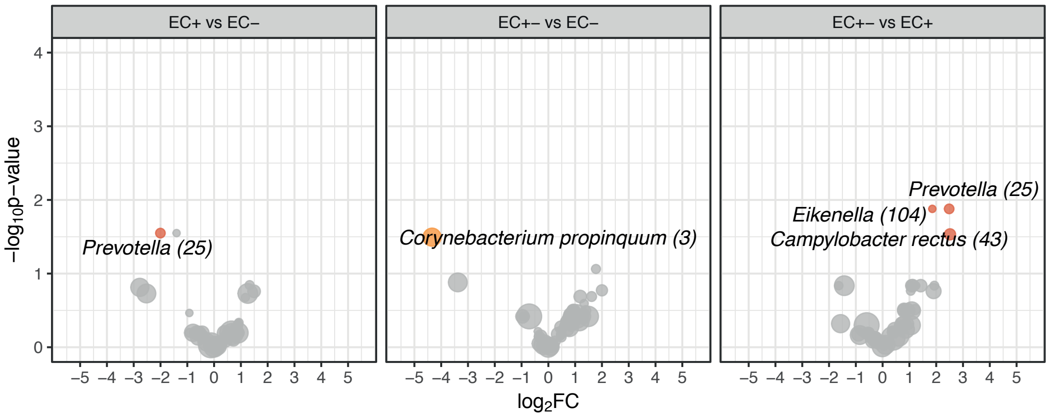

Supplementary Figure 7 – Differentially abundant OTUs at baseline between carriage$_3$ outcome phenotypes (metagenomeSeq). OTUs that were differentially abundant in high-dense carriers (EC+, *n*=49) vs non-carriers (EC-, *n*=40), low-dense carriers (EC+-, *n*=27) vs non-carriers and low-dense carriers vs high-dense carriers, after adjustment for multiple comparisons (Benjamini-Hochberg) were depicted using volcano plots. OTUs are shown as data points, coloured according to the carriage$_3$ outcome they are associated with and labeled. Non-significant OTUs are depicted as gray data points and are not labeled. The size of the data points corresponds with the mean relative abundance within that group. The plot depicts the relationship between effect size (log$_2$-fold change between groups) and significance (–log$_{10}$-transformed p-values) for each of these OTUs. Data were obtained by metagenomeSeq analysis. Data were not stratified by vaccine. See also Supplementary Table 6.

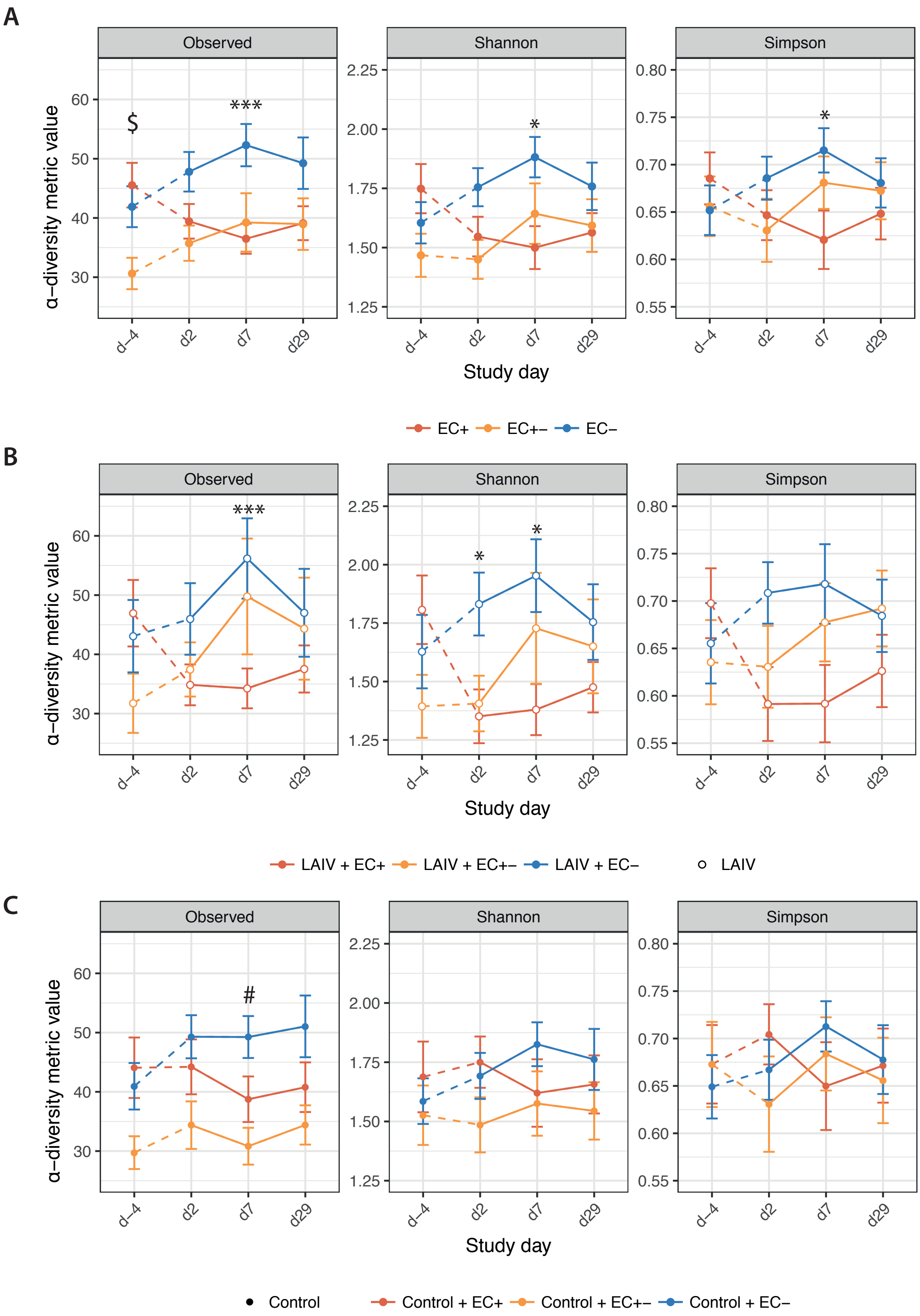

* EC+ vs EC-
\# EC+- vs EC-
$ EC+- vs EC+

Supplementary Figure 8 – -diversity measures stratified by carriage$_3$ outcome and vaccine. We tested the number of observed species, Simpson and Shannon diversity indices. Points represent mean values and whiskers depict the standard error of the mean. A non-stratified analysis **(A)** and stratified analysis for LAIV **(B)** and controls **(C)** were shown. P-values were derived from mixed linear models with subject as random effect. Significant differences between high-dense and non-carriers, between low-dense and non-carriers and between low-dense and high-dense carriers were denoted with *, # and $, respectively. One symbol, $p<0.05$; two symbols, $0.005 \leq p < 0.01$ and three symbols $p<0.005$. Red, EC+, high-dense carriers; blue, EC-, non-carriers and orange, EC+-, low-dense carriers. See Supplementary Table 7 for sample size per study day/carriage$_3$ outcome/vaccine (sample size is the same for each -diversity measure tested).

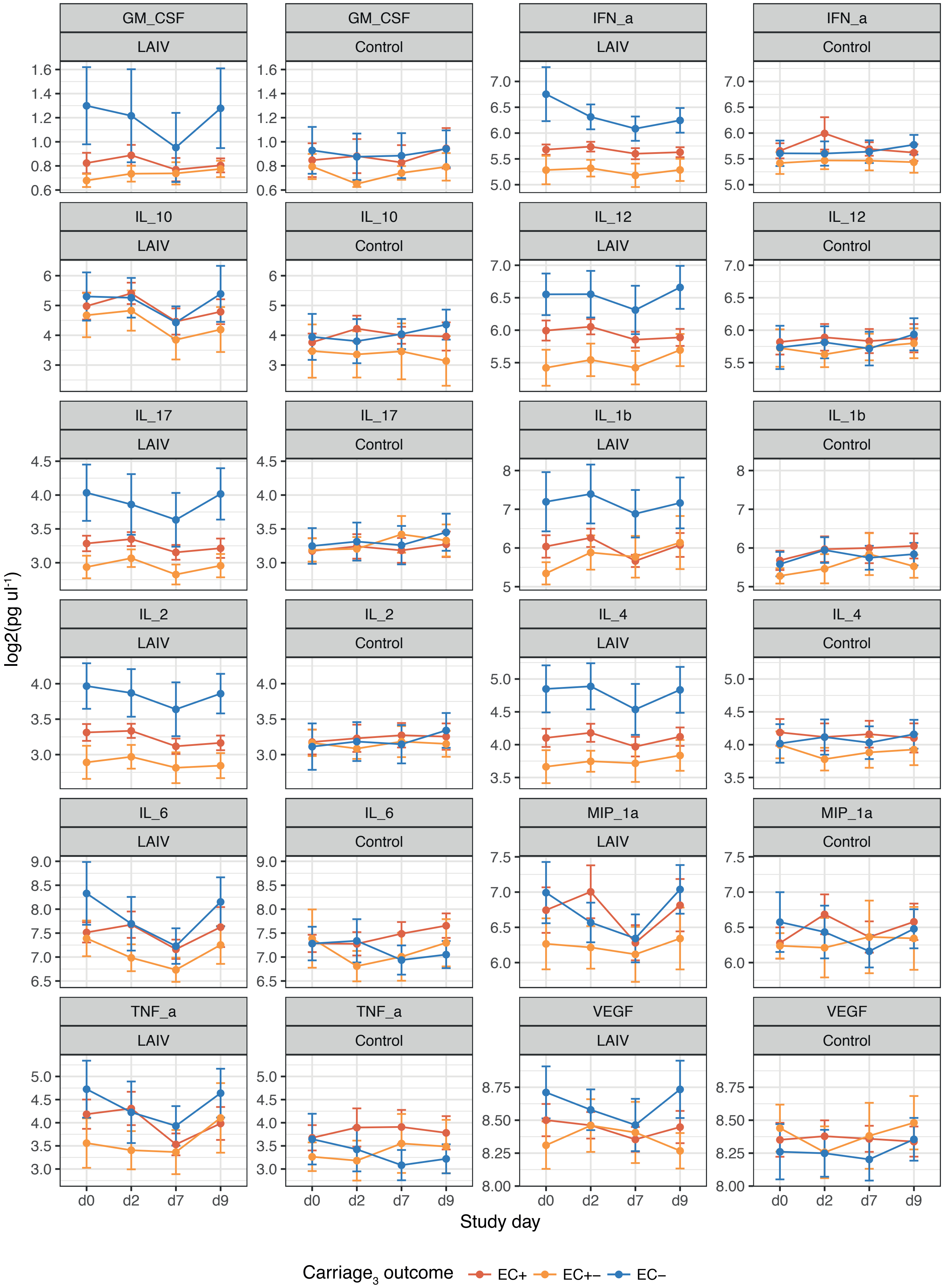
GM_CSF
LAIV
GM_CSF
Control
IFN_a
LAIV
IFN_a
Control
IL_10
LAIV
IL_10
Control
IL_12
LAIV
IL_12
Control
IL_17
LAIV
IL_17
Control
IL_1b
LAIV
IL_1b
Control
IL_2
LAIV
IL_2
Control
IL_4
LAIV
IL_4
Control
IL_6
LAIV
IL_6
Control
MIP_1a
LAIV
MIP_1a
Control
TNF_a
LAIV
TNF_a
Control
VEGF
LAIV
VEGF
Control
log2(pg ul-1)
d0 d2 d7 d9
Study day
Carriage3 outcome
EC+
EC+−
EC−

Supplementary Figure 9 – Cytokine dynamics in relation to pneumococcal carriage$_3$ outcome, stratified by vaccine group. Cytokine concentrations were selected for 12 cytokines after baseline (i.e. at day 0, 2, 7 and 9). Means (points) and standard errors of the mean were (whiskers) of log_2-transformed cytokine levels in (pg ml^{-1}) were plotted at each time point and stratified by carriage$_3$ outcome. Separate plots were generated for the LAIV and control groups. For statistical assessment, see Supplementary Table 8. Red, EC+, high-dense carriers; blue (*n*=18 for LAIV and *n*=18 for controls for each cytokine and at each time point), EC-, non-carriers (*n*=7 for LAIV and *n*=12 for controls) and orange, EC+-, low-dense carriers (*n*=9 for LAIV and *n*=8 for controls).

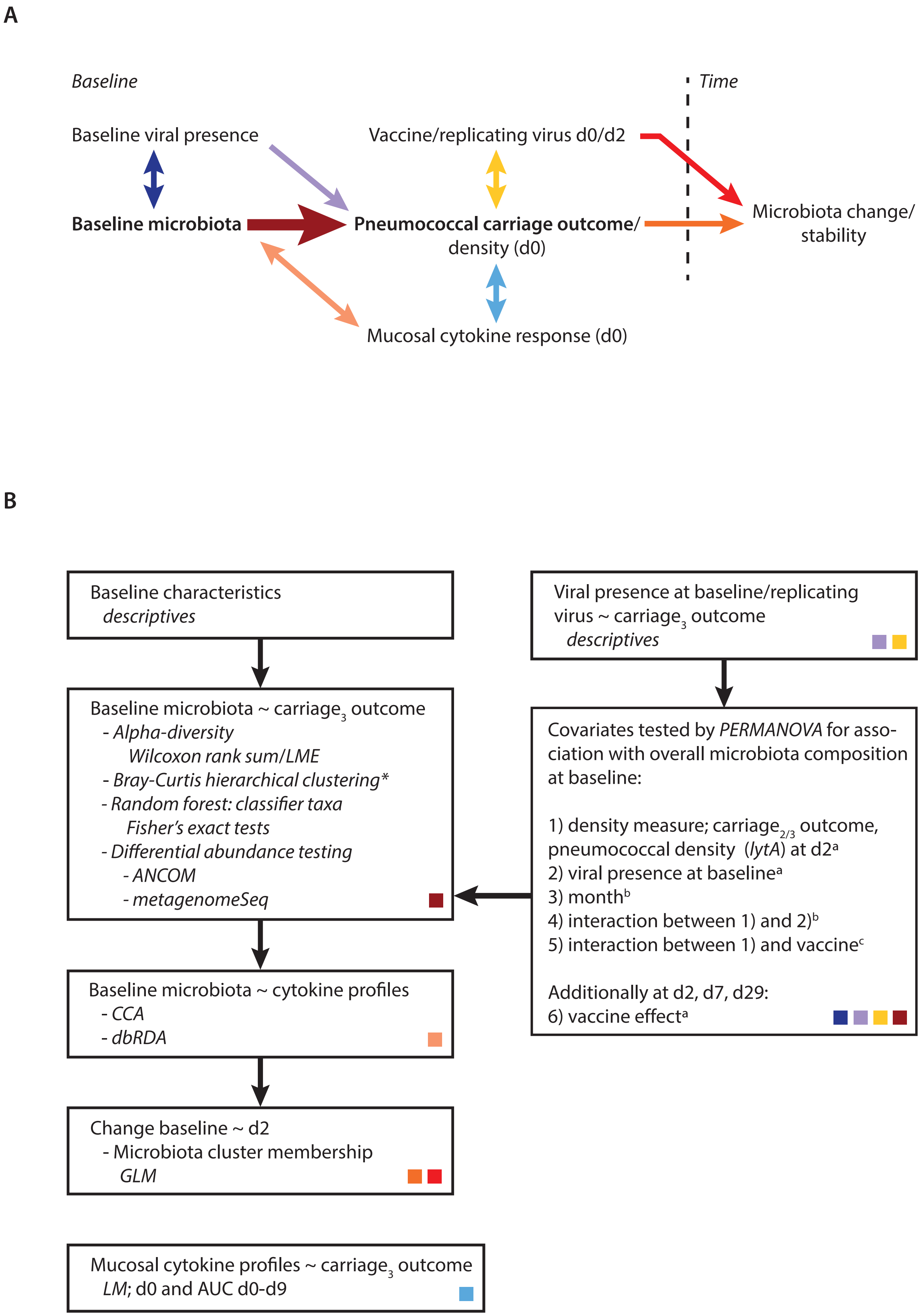
A
Baseline
Time
Baseline viral presence
Vaccine/replicating virus d0/d2
Baseline microbiota
Pneumococcal carriage outcome/ density (d0)
Microbiota change/ stability
Mucosal cytokine response (d0)
B
Baseline characteristics
descriptives
Viral presence at baseline/replicating virus ~ carriage$_3$ outcome
descriptives
Baseline microbiota ~ carriage$_3$ outcome
- Alpha-diversity
Wilcoxon rank sum/LME
- Bray-Curtis hierarchical clustering*
- Random forest: classifier taxa
Fisher's exact tests
- Differential abundance testing
- ANCOM
- metagenomeSeq
Covariates tested by PERMANOVA for association with overall microbiota composition at baseline:
1) density measure; carriage$_{2/3}$ outcome, pneumococcal density (lytA) at d2[a]
2) viral presence at baseline[a]
3) month[b]
4) interaction between 1) and 2)[b]
5) interaction between 1) and vaccine[c]
Additionally at d2, d7, d29:
6) vaccine effect[a]
Baseline microbiota ~ cytokine profiles
- CCA
- dbRDA
Change baseline ~ d2
- Microbiota cluster membership
GLM
Mucosal cytokine profiles ~ carriage$_3$ outcome
LM; d0 and AUC d0-d9

Supplementary Figure 10 – Schematic on primary/secondary research questions explored and data analysis flow chart. (A) Schematic providing an overview of all primary/secondary research questions explored between microbiota, pneumococcal carriage (density) and mucosal cytokine data explored in the paper. The thickest arrow represents the primary research question, i.e. what is the association between baseline nasal microbiota composition and pneumococcal $carriage_3$ outcome. Arrows are color coded and correspond with panel **(B)**, where we provide an overview of the statistical techniques (in *italics*) used to explore each question/association. ~ denotes the associations explored. Associations between (overall) microbiota composition and specific covariates were extensively explored. These covariates were tested because [a]the covariate was a variable of interest, [b]associations between this covariate and nasal/nasopharyngeal microbiota are described in literature or [c]this covariate was demonstrated to impact other variables based on previous work from our group[1,2]. * including *post-hoc* stratified clustering analysis. d, day; CCA, canonical correspondence analyses; dbRDA, distance-based redundancy analyses; LME, linear mixed-effects model; LM, linear model; GLM, generalized linear model; PERMANOVA, permutational analysis of variance.

SUPPLEMENTARY TABLES

Supplementary Table 1 - Baseline tables

(A) Baseline characteristics of the participants stratified by study and vaccination.

	Overall	LAIV	Control
n	117	55	62
Median age (range) - yr	20.0 (18.0, 48.0)	20.0 (18.0, 34.0)	20.0 (18.0, 48.0)
Female - no. (%)	68 (58.1)	36 (65.5)	32 (51.6)
Median dose (range) - CFU/nostril	76333 (51000, 88000)	74500 (51000, 88000)	77250 (51000, 88000)
Time between vaccination and inoculation (mean (sd))	3.0 (0.1)	3.0 (0.1)	3.0 (0.1)

(B) Baseline characteristics of the participants stratified by carriage$_3$ outcome.

	EC+	EC+-	EC-
n	49	27	41
Median age (range) - yr	20.0 (18.0, 48.0)	19.0 (18.0, 41.0)	20.0 (18.0, 34.0)
Female - no. (%)	31 (63.3)	13 (48.1)	24 (58.5)
Median dose (range) - CFU/nostril	76333 (51000, 88000)	76333 (51000, 88000)	77000 (55667, 86000)
Time between vaccination and inoculation (mean (sd))	3.0 (0.1)	3.0 (0.0)	3.0 (0.2)

(C) Baseline characteristics of the participants stratified by vaccination and carriage$_3$ outcome.

	LAIV			Control		
	EC+	EC+-	EC-	EC+	EC+-	EC-
n	25	12	18	24	15	23
Median age (range) - yr	20.0 (18.0, 25.0)	19.0 (18.0, 23.0)	20.0 (18.0, 34.0)	20.0 (18.0, 48.0)	20.0 (18.0, 41.0)	20.0 (18.0, 29.0)
Female - no. (%)	16 (64.0)	7 (58.3)	13 (72.2)	15 (62.5)	6 (40.0)	11 (47.8)
Median dose (range) - CFU/nostril	70833 (51000, 86333)	73750 (67333, 88000)	79416 (55667, 86000)	78167 (51000, 88000)	76333 (51000, 86000)	77000 (55667, 86000)
Time between vaccination and inoculation (mean (sd))	3.0 (0.0)	3.0 (0.0)	3.1 (0.2)	3.0 (0.2)	3.0 (0.0)	3.0 (0.0)

yr, year; EC+, high-dense carriers; EC-, non-carriers and EC+-, low-dense carriers.

Supplementary Table 2 - Nasal wash sample availability

Time point	LAIV	Control	Overall
d-4	55 (100)	61 (98)	116 (99)
d2	54 (98)	60 (97)	114 (97)
d7	54 (98)	62 (100)	116 (99)
d29	50 (91)	55 (89)	105 (90)

Percentages correspond with the proportion of samples available at each time point per vaccine group, out of the total number of participants in that group.

Supplementary Table 3 – Associations between the baseline nasal bacterial community composition and pneumococcal carriage outcome.

(A) Associations baseline nasal microbiota and $carriage_2$ outcome (based on culturing results).

Variable	Df	SumsOfSqs	MeanSqs	F.Model	R^2	p-value
$Carriage_2$ outcome	1	0,33	0,33	1,25	1,09%	0,260
Month	4	1,07	0,27	1,01	3,52%	0,433
Any virus at baseline	1	0,13	0,13	0,50	0,44%	0,872
$Carriage_2$ outcome:Vaccine	2	0,58	0,29	1,09	1,90%	0,349
$Carriage_2$ outcome:Any virus at baseline	1	0,44	0,44	1,65	1,44%	0,117
Residuals	105	27,80	0,26		91,61%	
Total	114	30,34			100,00%	

(B) Associations baseline nasal microbiota and pneumococcal density (based on lytA; log_{10}+1-transformed values).

Variable	Df	SumsOfSqs	MeanSqs	F.Model	R^2	p-value
Day 2 pneumococcal density (lytA)	1	0,51	0,51	1,95	1,69%	0,076
Month	4	1,06	0,27	1,01	3,50%	0,452
Any virus at baseline	1	0,14	0,14	0,51	0,45%	0,836
Day 2 pneumococcal density (lytA):Vaccine	1	0,43	0,43	1,62	1,41%	0,127
Day 2 pneumococcal density (lytA):Any virus at baseline	1	0,26	0,26	0,99	0,86%	0,404
Residuals	106	27,95	0,26		92,10%	
Total	114	30,34			100,00%	

The effect of pneumococcal density/$carriage_2$ outcome was adjusted for month, presence of any virus at baseline (day -4), month of sampling (i.e. seasonal effects) and the interactions between $carriage_2$ outcome/pneumococcal density and vaccine/presence of any virus at baseline. These interactions were included to properly assess the associations between baseline microbiota and $carriage_2$ outcome/pneumococcal density, the latter of which could have been impacted by viral presence. Analyses were performed using PERMANOVA. For a detailed assessment on the association between baseline nasal microbiota and $carriage_3$ outcome, see Table 1.

Supplementary Table 4 - Viral co-infection rates in oropharyngeal and nasosorbtion samples.

(A) Influenzavirus detected at each time point in the oropharynx

study_day	Infl_A	Infl_B	Infl_AB
d-4	1/115 (0.9%)	0/115 (0.0%)	1/115 (0.9%)
d2	3/54 (5.6%)	0/54 (0.0%)	3/54 (5.6%)
d7	0/54 (0.0%)	0/54 (0.0%)	0/54 (0.0%)
d29	0/49 (0.0%)	0/49 (0.0%)	0/49 (0.0%)

(B) Respiratory viruses detected at baseline in the oropharynx

Virus	EC+	EC+-	EC-	overall
Any virus	4/48 (8.3%)	1/27 (3.7%)	6/40 (15.0%)	11/115 (9.6%)
Human rhinovirus	2/48 (4.2%)	0/27 (0.0%)	1/40 (2.5%)	3/115 (2.6%)
Enterovirus	0/48 (0.0%)	0/27 (0.0%)	0/40 (0.0%)	0/115 (0.0%)
Human bocavirus	0/48 (0.0%)	1/27 (3.7%)	0/40 (0.0%)	1/115 (0.9%)
Human coronaviruses (pooled)	0/48 (0.0%)	0/27 (0.0%)	2/40 (5.0%)	2/115 (1.7%)
OC43	0/49 (0.0%)	0/27 (0.0%)	0/40 (0.0%)	0/116 (0.0%)
NL63	0/49 (0.0%)	0/27 (0.0%)	0/40 (0.0%)	0/116 (0.0%)
229E	0/49 (0.0%)	0/27 (0.0%)	1/40 (2.5%)	1/116 (0.9%)
Parainfluenza viruses 1/3 (pooled)	0/48 (0.0%)	0/27 (0.0%)	0/40 (0.0%)	0/115 (0.0%)
Type 1	0/49 (0.0%)	0/27 (0.0%)	0/40 (0.0%)	0/116 (0.0%)
Type 3	0/49 (0.0%)	0/27 (0.0%)	0/40 (0.0%)	0/116 (0.0%)
Parainfluenza viruses 2/4 (pooled)	0/48 (0.0%)	0/27 (0.0%)	1/40 (2.5%)	1/115 (0.9%)
Type 2	0/49 (0.0%)	0/27 (0.0%)	1/40 (2.5%)	1/116 (0.9%)
Type 4	0/49 (0.0%)	0/27 (0.0%)	0/40 (0.0%)	0/116 (0.0%)
Human adenovirus	0/48 (0.0%)	0/27 (0.0%)	0/40 (0.0%)	0/115 (0.0%)
Respiratory syncytial virus A	2/48 (4.2%)	0/27 (0.0%)	0/40 (0.0%)	2/115 (1.7%)
Respiratory syncytial virus B	0/48 (0.0%)	0/27 (0.0%)	1/40 (2.5%)	1/115 (0.9%)
Human metapneumovirus	0/48 (0.0%)	0/27 (0.0%)	0/40 (0.0%)	0/115 (0.0%)

(C) Influenzavirus detected at day 0 and day 2 in Nasosorption samples

study_day	Infl_A	Infl_B	Infl_AB
d0	10/53 (18.9%)	10/53 (18.9%)	17/53 (32.1%)
d2	2/54 (3.7%)	7/54 (13.0%)	9/54 (16.7%)

(D) Influenzavirus A/B detecected at day 0 and/or day 2 in Nasosorption samples, stratified by carriage status$_3$ outcome.

Virus	EC+	EC+-	EC-
Human influenzavirus A/B	7/21 (33.3%)	2/10 (20.0%)	6/16 (37.5%)

(E) Influenzavirus A/B detecected at day 0 and/or day 2 in nasosorbtion samples, stratified by carriage status$_3$ outcome, excluding those individuals with presence of any repiratory virus at baseline.

Virus	EC+	EC+-	EC-
Human influenzavirus A/B	6/18 (33.3%)	2/10 (20.0%)	4/14 (28.6%)

Supplementary Table 5 - Number of samples in each cluster at each time point, stratified by carriage$_3$ outcome.

(A) High-dense carriers.

Cluster	d-4	d2	d7	d29
STA	17	13	10	11
CDG	11	10	15	14
COR	11	10	12	9
HPH	1	1	0	3
MOR	0	2	0	1
PEP/MIX	1	2	1	1
FUS	1	0	0	0
STR	1	4	6	1

(B) Low-dense carriers.

Cluster	d-4	d2	d7	d29
STA	5	5	3	5
CDG	10	10	7	9
COR	8	8	10	4
HPH	2	1	3	1
MOR	1	1	1	0
PEP/MIX	0	1	1	2
FUS	1	1	1	1
STR	0	0	0	0

(C) Non-carriers.

Cluster	d-4	d2	d7	d29
STA	16	16	17	14
CDG	4	3	4	7
COR	9	7	9	5
HPH	1	3	3	1
MOR	4	4	1	2
PEP/MIX	1	2	2	3
FUS	2	3	2	2
STR	0	0	0	0

Clusters were characterized by *Staphylococcus* (2) (STA); *Corynebacterium* (3) and *Dolosigranulum* (4) spp. (CDG); *Corynebacterium* (1; COR); *Haemophilus* (9; HPH), *Moraxella* (6; MOR), *Fusobacterium* (10; FUS), *Streptococcus* (7; STR), and *Peptoniphilus* (5), *Finegoldia* (8), *Anaerococcus* (11) and *Streptococcus salivarius* (13; PEP/MIX).

Supplementary Table 6 - Statistically significant differences bacterial community members at baseline between carriage status$_3$ outcome.

OTU	logFC	P.Value	adj.P.Val	-log10.adj.P.val	contrast	group_sig
**Prevotella (25)***	-2,00	1,39E+00	2,82E-02	1,55	EC+ vs EC-	EC+
**Corynebacterium propinquum (3)**	-4,34	5,75E+00	3,20E-02	1,49	EC+- vs EC-	EC+-
**Eikenella (104)**	1,85	9,57E-01	1,32E-02	1,88	EC+- vs EC+	EC+
**Prevotella (25)***	2,48	1,39E+00	1,32E-02	1,88	EC+- vs EC+	EC+
**Campylobacter rectus (43)**	2,51	1,86E+00	2,93E-02	1,53	EC+- vs EC+	EC+

Only OTUs with an adjusted P-value of 0.05 and a $-1.5 < \log_2FC < 1.5$ were shown. Asterisks denote those OTUs that were selected more than once (i.e. these OTUs were significantly different in more than one contrast). The 'group_sig' column denotes the carriage status outcome (EC+, high-dense carriers; EC-, non-carriers and EC+-, low-dense carriers) a specific OTU was positively associated with. Tests were not stratified for vaccine group. logFC, $\log_2$FC; adj.P.val, Benjamini-Hochberg adjusted p-values.

Supplementary Table 7 - Sample size for α-diversity measure comparisons by study day, $carriage_3$ outcome and vaccine.

(A) Sample size Supplementary Figure 8A.

study_day	EC+	EC+-	EC-
d-4	49	27	40
d2	47	27	40
d7	48	27	41
d29	43	24	38

(B) Sample size Supplementary Figure 8B.

study_day	vaccine	EC+	EC+-	EC-
d-4	LAIV	25	12	18
d2	LAIV	24	12	18
d7	LAIV	24	12	18
d29	LAIV	22	11	17

(C) Sample size Supplementary Figure 8C.

study_day	vaccine	EC+	EC+-	EC-
d-4	Control	24	15	22
d2	Control	23	15	22
d7	Control	24	15	23
d29	Control	21	13	21

See also Supplementary Figure 8. EC+, high-dense carriers; EC-, non-carriers and EC+-, low-dense carriers.

Supplementary Table 8 - Cytokine levels related to carriage$_3$ outcome, stratified by vaccine.

(A) Cytokine levels at day 0.

cytokine	contrast	p.value	coef
**IFN_a**	LAIV: EC+ vs EC-	0,006	-1,074
**IL_17**	LAIV: EC+ vs EC-	0,060	-0,750
**IL_1b**	LAIV: EC+ vs EC-	0,076	-1,155
**IL_4**	LAIV: EC+ vs EC-	0,098	-0,746
**GM_CSF**	LAIV: EC+- vs EC-	0,057	-0,620
**IFN_a**	LAIV: EC+- vs EC-	0,001	-1,469
**IL_12**	LAIV: EC+- vs EC-	0,028	-1,131
**IL_17**	LAIV: EC+- vs EC-	0,011	-1,098
**IL_1b**	LAIV: EC+- vs EC-	0,007	-1,849
**IL_2**	LAIV: EC+- vs EC-	0,017	-1,076
**IL_4**	LAIV: EC+- vs EC-	0,012	-1,185

(B) Area-under-the-curves (AUCs).

cytokine	contrast	p.value	coef
**IFN_a**	LAIV: EC+ vs EC-	0,068	-5,410
**IL_1b**	LAIV: EC+ vs EC-	0,075	-10,467
**IFN_a**	LAIV: EC+- vs EC-	0,004	-9,070
**IL_12**	LAIV: EC+- vs EC-	0,023	-8,748
**IL_17**	LAIV: EC+- vs EC-	0,045	-7,767
**IL_1b**	LAIV: EC+- vs EC-	0,068	-12,054
**IL_2**	LAIV: EC+- vs EC-	0,028	-8,128
**IL_4**	LAIV: EC+- vs EC-	0,021	-9,046

All results with $p < 0.1$ shown. p-values $0.05 \le p < 0.1$ marked in gray, p-values < 0.05 marked in black. EC+, high-dense carriers; EC-, non-carriers and EC+-, low-dense carriers.

Supplementary Table 9 - Overview primers.

Target	Forward (5' - 3')	Reverse (5' - 3')	Reference
**16S (quantification)**	CGA AAG CGT GGG GAG CAA A	GTT CGT ACT CCC CAG GCG G	Bogaert *et al*. PLoS ONE. 2011.[7]
**16S V4 (533F/806R)**	GTGCCAGCAGCCGCGGTAA	ATTAGATACCCTGGTAGTCC	Weisenburg *et al*. J Bacteriol. 1991.
			Alm *et al*. Appl Environ Microbiol. 1996.
**Human rhinovirus**	CGA AGA GTC TAC TGT GCT CAC CTT	GTA GTC GGT CCC ATC CC	Hoek *et al*. Scan J Infect Dis. 2012.[19]
	GAA GAT CCT ATT GCG CTT AGC TGT		
	GGT GTG AAG AGC CCC GTG TG		
	GGT GTG AAG ACT CGC ATG TGC		
**Enterovirus**	GAC ATG GTG YGA AGA GTC TAT TGA	GAT TGT CAC CAT AAG CAG CCA	Hoek *et al*. Scan J Infect Dis. 2012.[19]
**Human bocavirus**	GGA AGA GAC ACT GGC AGA CAA	GGG TGT TCC TGA TGA TAT GAG C	Allander *et al*. Clin Infect Dis. 2007.[13]
**Human coronaviruses**			
**OC43**	CGA TGA GGC TAT TCC GAC TAG GT	CCT TCC TGA GCC TTC AAT ATA GTA ACC	Elden *et al*. J Clin Microbiol. 2004.[14]
**NL63**	GCG TGT TCC TAC CAG AGA GGA	GCT GTG GAA AAC CTT TGG CA	Zlateva *et al*. Arch Virol. 2012.[15]
**229E**	CAG TCA AAT GGG CTG ATG CA	CAA AGG GCT ATA AAG AGA ATA AGG TAT TCT	Elden *et al*. J Clin Microbiol. 2004.[14]
**Parainfluenza viruses 1-4**			
**Type 1**	TGA TTT AAA CCC GGT AAT TTC TCA T	CCT TGT TCC TGC AGC TAT TAC AGA	Van de Pol *et al*. J Clin Microbiol. 2007.[12]
**Type 2**	AGG ACT ATG AAA ACC ATT TAC CTA AGT [illegible]	AAG CAA GTC TCA GTT CAG CTA GAT CA	Van de Pol *et al*. J Clin Microbiol. 2007.[12]
**Type 3**	TGA TGA AAG ATC AGA TTA TGC ATA TC	CCG GGA CAC CCA GTT GTG	Van de Pol *et al*. J Clin Microbiol. 2007.[12]
		CCA GGA CAC CCA GTT GTG	
**Type 4**	CAA AYG ATC CAC AGC AAA GAT TC	ATG TGG CCT GTA AGG AAA GCA	Van de Pol *et al*. J Clin Microbiol. 2007.[12]
**Human adenovirus**	TTT GAG GTG GAY CCM ATG GA	AGA ASG GSG TRC GCA GGT A	Van de Pol *et al*. J Clin Microbiol. 2007.[12]
	TTT GAG GTY GAY CCC ATG GA	AGA ASG GTG TRC GCA GAT A	
**Respiratory syncytial virus A**	AGA TCA ACT TCT GTC ATC CAG CAA	TTC TGC ACA TCA TAA TTA GGA GTA TCA AT	Elden *et al*. J Clin Microbiol. 2003/2005.[16,17]
**Respiratory syncytial virus B**	AAG ATG CAA ATC ATA AAT TCA CAG GA	TGA TAT CCA GCA TCT TTA AGT ATC TTT ATA G[illegible]	Elden *et al*. J Clin Microbiol. 2003/2005.[16,17]
**Human metapneumovirus**	CAT GCC CAC TAT AAA AGG TCA G	CAC CCC AGT CTT TCT TGA AA	Maertzdorf *et al*. J Clin Microbiol. 2004.[18]
**Influenza A**	CTT CTR ACC GAG GTC GAA ACG TA	TCT TGT CTT TAG CCA YTC CAT GAG	Hoek *et al*. Scan J Infect Dis. 2012.[19]
**Influenza B**	GAG ACA CAA TTG CCT ACC TGC TT	TTC TTT CCC ACC GAA CCA AC	Ward *et al*. J Clin Virolog. 2004.[20]

SUPPLEMENTARY REFERENCES

1. Rylance, J. *et al.* Effect of live-attenuated influenza vaccine on pneumococcal carriage. *biorxiv.org* (2018). doi:10.1101/343319
2. Rylance, J. *et al.* Two Randomized Trials of Effect of Live Attenuated Influenza Vaccine on Pneumococcal Colonization. *Am J Respir Crit Care Med* **199,** 1160–1163 (2019).
3. Gritzfeld, J. F. *et al.* Experimental human pneumococcal carriage. *J Vis Exp* e50115–e50115 (2013). doi:10.3791/50115
4. Collins, A. M. *et al.* First Human Challenge Testing of a Pneumococcal Vaccine. Double-Blind Randomized Controlled Trial. *Am J Respir Crit Care Med* **192,** 853–858 (2015).
5. Ferreira, D. M. *et al.* Controlled human infection and rechallenge with Streptococcus pneumoniae reveals the protective efficacy of carriage in healthy adults. *Am J Respir Crit Care Med* **187,** 855–864 (2013).
6. Wyllie, A. L. *et al.* Streptococcus pneumoniae in Saliva of Dutch Primary School Children. *PLoS ONE* **9,** e102045 (2014).
7. Bogaert, D. *et al.* Variability and diversity of nasopharyngeal microbiota in children: a metagenomic analysis. *PLoS ONE* **6,** e17035 (2011).
8. Biesbroek, G. *et al.* Early Respiratory Microbiota Composition Determines Bacterial Succession Patterns and Respiratory Health in Children. *Am J Respir Crit Care Med* **190,** 1283–1292 (2014).
9. Carvalho, M. D. G. S. *et al.* Evaluation and improvement of real-time PCR assays targeting lytA, ply, and psaA genes for detection of pneumococcal DNA. *J Clin Microbiol* **45,** 2460–2466 (2007).
10. Trzciński, K. *et al.* Superiority of Trans-Oral over Trans-Nasal Sampling in Detecting Streptococcus pneumoniae Colonization in Adults. *PLoS ONE* **8,** e60520 (2013).
11. van Elden, L. J., Nijhuis, M., Schipper, P., Schuurman, R. & van Loon, A. M. Simultaneous detection of influenza viruses A and B using real-time quantitative PCR. *J Clin Microbiol* **39,** 196–200 (2001).
12. van de Pol, A. C. *et al.* Increased detection of respiratory syncytial virus, influenza viruses, parainfluenza viruses, and adenoviruses with real-time PCR in samples from patients with respiratory symptoms. *J Clin Microbiol* **45,** 2260–2262 (2007).
13. Allander, T. *et al.* Human Bocavirus and Acute Wheezing in Children. *Clin Infect Dis* **44,** 904–910 (2007).
14. van Elden, L. J. R. *et al.* Frequent detection of human coronaviruses in clinical specimens from patients with respiratory tract infection by use of a novel real-time reverse-transcriptase polymerase chain reaction. *J Infect Dis* **189,** 652–657 (2004).
15. Zlateva, K. T. *et al.* No novel coronaviruses identified in a large collection of human nasopharyngeal specimens using family-wide CODEHOP-based primers. *Arch Virol* **158,** 251–255 (2012).
16. van Elden, L. J. R. *et al.* Applicability of a Real-Time Quantitative PCR Assay for Diagnosis of Respiratory Syncytial Virus Infection in Immunocompromised Adults. *J Clin Microbiol* **41,** 4378–4381 (2003).
17. van Elden, L. J. R. *et al.* Applicability of a Real-Time Quantitative PCR Assay for Diagnosis of Respiratory Syncytial Virus Infection in Immunocompromised Adults. *J Clin Microbiol* **43,** 4308–4308 (2005).
18. Maertzdorf, J. *et al.* Real-Time Reverse Transcriptase PCR Assay for Detection of Human Metapneumoviruses from All Known Genetic Lineages. *J Clin Microbiol* **42,** 981–986 (2004).
19. Hoek, R. A. S. *et al.* Incidence of viral respiratory pathogens causing exacerbations in adult cystic fibrosis patients. *Scand J Infect Dis* **45,** 65–69 (2013).
20. Ward, C. L. *et al.* Design and performance testing of quantitative real time PCR assays for influenza A and B viral load measurement. *J. Clin. Virol.* **29,** 179–188 (2004).

21. Jochems, S. P. *et al.* Inflammation induced by influenza virus impairs human innate immune control of pneumococcus. *Nat Immunol* **388,** 3027 (2018).
22. Bosch, A. A. T. M. *et al.* Maturation of the Infant Respiratory Microbiota, Environmental Drivers, and Health Consequences. A Prospective Cohort Study. *Am J Respir Crit Care Med* **196,** 1582–1590 (2017).
23. Joshi, N. A. & Fass, J. N. Sickle: A sliding-window, adaptive, quality-based trimming tool for FastQ files.
24. Nikolenko, S. I., Korobeynikov, A. I. & Alekseyev, M. A. BayesHammer: Bayesian clustering for error correction in single-cell sequencing. *BMC Genomics* **14 Suppl 1,** S7 (2013).
25. Masella, A. P., Bartram, A. K., Truszkowski, J. M., Brown, D. G. & Neufeld, J. D. PANDAseq: paired-end assembler for illumina sequences. *BMC Bioinformatics* **13,** 31 (2012).
26. Schirmer, M. *et al.* Insight into biases and sequencing errors for amplicon sequencing with the Illumina MiSeq platform. *Nucleic Acids Res* **43,** e37–e37 (2015).
27. Caporaso, J. G. *et al.* QIIME allows analysis of high-throughput community sequencing data. *Nat Methods* **7,** 335–336 (2010).
28. Westcott, S. L. & Schloss, P. D. De novo clustering methods outperform reference-based methods for assigning 16S rRNA gene sequences to operational taxonomic units. *PeerJ* **3,** e1487 (2015).
29. Wang, Q., Garrity, G. M., Tiedje, J. M. & Cole, J. R. Naive Bayesian classifier for rapid assignment of rRNA sequences into the new bacterial taxonomy. *Appl Environ Microbiol* **73,** 5261–5267 (2007).
30. Claesson, M. J. *et al.* Comparative analysis of pyrosequencing and a phylogenetic microarray for exploring microbial community structures in the human distal intestine. *PLoS ONE* **4,** e6669 (2009).
31. Quast, C. *et al.* The SILVA ribosomal RNA gene database project: improved data processing and web-based tools. *Nucleic Acids Res* **41,** D590–6 (2013).
32. Subramanian, S. *et al.* Persistent gut microbiota immaturity in malnourished Bangladeshi children. *Nature* **510,** 417–421 (2014).
33. Mandal, S. *et al.* Analysis of composition of microbiomes: a novel method for studying microbial composition. *Microb Ecol Health Dis* **26,** 27663 (2015).
34. Paulson, J. N., Stine, O. C., Bravo, H. C. & Pop, M. Differential abundance analysis for microbial marker-gene surveys. *Nat Methods* **10,** 1200–1202 (2013).
35. Planer, J. D. *et al.* Development of the gut microbiota and mucosal IgA responses in twins and gnotobiotic mice. *Nature* (2016). doi:10.1038/nature17940
36. Salter, S. J. *et al.* Reagent and laboratory contamination can critically impact sequence-based microbiome analyses. *BMC Biol* **12,** 87 (2014).
37. Oksanen, J. *et al.* vegan: Community Ecology Package. (2015).
38. Hothorn, T., Bretz, F., Westfall, P. & Heiberger, R. M. Package 'multcomp'. (2016).
39. de Steenhuijsen Piters, W. A. A. *et al.* Nasopharyngeal Microbiota, Host Transcriptome, and Disease Severity in Children with Respiratory Syncytial Virus Infection. *Am J Respir Crit Care Med* **194,** 1104–1115 (2016).
40. Hennig, C. fpc: Flexible Procedures for Clustering. (2015).
41. Gesmann, M. & de Castillo, D. Using the Google Visualisation API with R. *R Journal* **3,** 40–44 (2011).
42. Clarke, K. R. Non-parametric multivariate analyses of changes in community structure. **18,** 117–143 (1993).
