# Supplementary material for: Interaction between the nasal microbiota and S. pneumoniae in the context of live-attenuated influenza vaccine
Source: Nat Commun. 2019 Jul 5;10:2981. doi: 10.1038/s41467-019-10814-9 (PMC6611866; doi:10.1038/s41467-019-10814-9)
Supplement: Supplementary file 2 — Reporting Summary [file 41467_2019_10814_MOESM2_ESM.pdf]

nature research

Corresponding author(s): WAASP

Last updated by author(s): 11-4-2019

Reporting Summary

Nature Research wishes to improve the reproducibility of the work that we publish. This form provides structure for consistency and transparency in reporting. For further information on Nature Research policies, see Authors & Referees and the Editorial Policy Checklist.

Statistics

For all statistical analyses, confirm that the following items are present in the figure legend, table legend, main text, or Methods section.

n/a	Confirmed	
☐	☒	The exact sample size (*n*) for each experimental group/condition, given as a discrete number and unit of measurement
☐	☒	A statement on whether measurements were taken from distinct samples or whether the same sample was measured repeatedly
☐	☒	The statistical test(s) used AND whether they are one- or two-sided<br>*Only common tests should be described solely by name; describe more complex techniques in the Methods section.*
☐	☒	A description of all covariates tested
☐	☒	A description of any assumptions or corrections, such as tests of normality and adjustment for multiple comparisons
☐	☒	A full description of the statistical parameters including central tendency (e.g. means) or other basic estimates (e.g. regression coefficient) AND variation (e.g. standard deviation) or associated estimates of uncertainty (e.g. confidence intervals)
☐	☒	For null hypothesis testing, the test statistic (e.g. *F*, *t*, *r*) with confidence intervals, effect sizes, degrees of freedom and *P* value noted<br>*Give P values as exact values whenever suitable.*
☒	☐	For Bayesian analysis, information on the choice of priors and Markov chain Monte Carlo settings
☐	☒	For hierarchical and complex designs, identification of the appropriate level for tests and full reporting of outcomes
☐	☒	Estimates of effect sizes (e.g. Cohen's *d*, Pearson's *r*), indicating how they were calculated

Our web collection on statistics for biologists contains articles on many of the points above.

Software and code

Policy information about availability of computer code

Data collection	Please see our Supplementary Information for a full list of software packages to process 16S-rRNA sequence data, including trimming, error correction, assembly and 97%-identity binning of reads into OTUs. In addition chimeric reads were removed and OTUs were taxonomically annotated using SILVA. Software packages included: Sickle, version 1.33; BayesHammer, SPAdes genome assembler toolkit, version 3.5.0; PANDAseq, version 2.9; Qiime version 1.9.1; VSEARCH version v2.0.3_linux_x86_64 and the Naïve Bayesian RDP classifier (version 2.2) using the SILVA 119 release reference database.
Data analysis	All stastical analyses were performed in the R version 3.3.0 within R studio version 0.99.902.

For manuscripts utilizing custom algorithms or software that are central to the research but not yet described in published literature, software must be made available to editors/reviewers. We strongly encourage code deposition in a community repository (e.g. GitHub). See the Nature Research guidelines for submitting code & software for further information.

Data

Policy information about availability of data

All manuscripts must include a data availability statement. This statement should provide the following information, where applicable:

- Accession codes, unique identifiers, or web links for publicly available datasets
- A list of figures that have associated raw data
- A description of any restrictions on data availability

The pre-processed, 16S-rRNA sequencing data generated during the current study are available from NCBI under bioproject accession number PRJNA421976. A reporting summary for this Article is available as a Supplementary Information file.

Field-specific reporting

Please select the one below that is the best fit for your research. If you are not sure, read the appropriate sections before making your selection.

☒ Life sciences ☐ Behavioural & social sciences ☐ Ecological, evolutionary & environmental sciences

For a reference copy of the document with all sections, see nature.com/documents/nr-reporting-summary-flat.pdf

Life sciences study design

All studies must disclose on these points even when the disclosure is negative.

Sample size	With baseline carriage rates of 50% (expected based on prior data from our model), 73 participants in each arm were required for 80% power to detect a 50% relative increase in pneumococcal acquisition at any time point, after 10% drop-out.
Data exclusions	Of 130 vaccinated volunteers, five were natural pneumococcal carriers (two in LAIV arm and three in control arm) and were excluded from further analysis. Another 8 subjects in the LAIV arm were excluded following a systematic LAIV dispensing error by a single practitioner, as recommended by the trial steering group. This resulted in a final 55 subjects analysed in the LAIV arm and 62 subjects in the control arm.
Replication	There was no cohort available with the same study design to replicate our results.
Randomization	Using a permuted-block algorithm (1:1, blocks of 10) held in sealed envelopes, participants were randomised to receive either nasal LAIV (Fluenz Tetra or FluMist Tetra, AstraZeneca, UK, used interchangeably due to procurement shortages) paired with intramuscular placebo (0.5ml normal saline), or nasal placebo [control] (0.2ml normal saline) paired with intramuscular Quadrivalent Inactivated Influenza Vaccination (Fluarix Tetra, GlaxoSmithKline, UK) (see supplemental methods for flu strains).
Blinding	This was a double-blinded, randomized trial where investigators were blinded during data collection and data analysis

Reporting for specific materials, systems and methods

We require information from authors about some types of materials, experimental systems and methods used in many studies. Here, indicate whether each material, system or method listed is relevant to your study. If you are not sure if a list item applies to your research, read the appropriate section before selecting a response.

Materials & experimental systems

n/a	Involved in the study	
☒	☐	Antibodies
☒	☐	Eukaryotic cell lines
☒	☐	Palaeontology
☒	☐	Animals and other organisms
☐	☒	Human research participants
☐	☒	Clinical data

Methods

n/a	Involved in the study	
☒	☐	ChIP-seq
☒	☐	Flow cytometry
☒	☐	MRI-based neuroimaging

Human research participants

Policy information about studies involving human research participants

Population characteristics	We recruited a cohort of participants who were enrolled in a single-centre, double-blinded, placebo-controlled trial (October 2015 - March 2016). We enrolled healthy, non-smoking individuals 17-48 years of age (median 20 years). 58% of included individuals were female and the average inoculation dose was 76,333 CFUs (range 51,000 - 88,000). See Supplementary Table 1 for details.<br>Exclusion criteria were: influenza or pneumococcal vaccination or clinically confirmed disease in the preceding two years; close contact with ‘high-risk’ individuals (children under 5, immunosuppressed, elderly); current febrile illness; use of antibiotics or immune-modulating medication; pregnancy.
Recruitment	Participants were recruited via poster, table displays with flyers and participant information sheets, or electronic advertisement. Posters and flyers were available in public places including notice boards in the Royal Liverpool University Hospital (RLUH), the Liverpool School of Tropical Medicine, local Universities and on public notice boards in the local area. We did also use social media (Facebook and twitter) and other media advertisement such as local newspapers, radio and television to reach study subjects. Interested parties were invited to contact a member of the clinical research team by phone and email. Our recruitment approach let to the inclusion of healthy young individuals, which is clearly stated in the main manuscript. Findings therefore only apply to this study group and care should be taken to extrapollate these results to high risk groups.
Ethics oversight	Ethical approval was granted by the Liverpool East NHS Research Committee (14-NW-1460), and all participants gave written informed consent.

Note that full information on the approval of the study protocol must also be provided in the manuscript.

Clinical data

Policy information about clinical studies

All manuscripts should comply with the ICMJE guidelines for publication of clinical research and a completed CONSORT checklist must be included with all submissions.

Clinical trial registration	The study was pre-registered (EudraCT 2014-004634-26).
Study protocol	https://www.clinicaltrialsregister.eu/ctr-search/trial/2014-004634-26/GB#B
Data collection	Patients were recruited and data were collected between October 2015 and March 2016.
Outcomes	We here tested two carriage outcome variables on the basis of nasal washes from day 2, 7 and 9: 1) carriage2 outcome (based on pneumococcal detection using conventional culture only), ‘carriers’, with a culture positive sample at any point and ‘non-carriers’, who were culture-negative at all times; and 2) carriage3 outcome (combination of pneumococcal detection using both conventional culture and molecular techniques), coded as ‘high-dense carriers’ (culture-positive at any point), ‘low-dense carriers’ (qPCR-positive and culture-negative) and ‘non-carriers’ (qPCR- and culture-negative at every point). Initial explorative analyses demonstrated higher explanatory power of carriage3 outcome, i.e. the variable incorporating qPCR results. We therefore decided to use this outcome variable throughout the rest of the manuscript instead of carriage2 outcome.
